# CRISPR/Cas9‐mediated knockout and overexpression studies reveal a role of maize phytochrome C in regulating flowering time and plant height

**DOI:** 10.1111/pbi.13429

**Published:** 2020-07-02

**Authors:** Quanquan Li, Guangxia Wu, Yongping Zhao, Baobao Wang, Binbin Zhao, Dexin Kong, Hongbin Wei, Cuixia Chen, Haiyang Wang

**Affiliations:** ^1^ State Key Laboratory of Crop Biology College of Agronomy Shandong Agricultural University Tai’an China; ^2^ Biotechnology Research Institute Chinese Academy of Agricultural Sciences Beijing China; ^3^ State Key Laboratory for Conservation and Utilization of Subtropical Agro‐Bioresources South China Agricultural University Guangzhou China; ^4^ Guangdong Laboratory for Lingnan Modern Agriculture Guangzhou China

**Keywords:** maize, phytochrome C, flowering time, shade‐avoidance syndrome, high‐density planting

## Abstract

Maize is a major staple crop widely used for food, feedstocks and industrial products. Shade‐avoidance syndrome (SAS), which is triggered when plants sense competition of light from neighbouring vegetation, is detrimental for maize yield production under high‐density planting conditions. Previous studies have shown that the red and far‐red photoreceptor phytochromes are responsible for perceiving the shading signals and triggering SAS in Arabidopsis; however, their roles in maize are less clear. In this study, we examined the expression patterns of *ZmPHYC1* and *ZmPHYC2* and found that *ZmPHYC1*, but not *ZmPHYC2*, is highly expressed in leaves and is regulated by the circadian clock. Both ZmPHYC1 and ZmPHYC2 proteins are localized to both the nucleus and cytoplasm under light conditions and both of them can interact with themselves or with ZmPHYBs. Heterologous expression of *ZmPHYCs* can complement the Arabidopsis *phyC‐2* mutant under constant red light conditions and confer an attenuated SAS in Arabidopsis in response to shading. Double knockout mutants of *ZmPHYC1* and *ZmPHYC2* created using the CRISPR/Cas9 technology display a moderate early‐flowering phenotype under long‐day conditions, whereas *ZmPHYC2* overexpression plants exhibit a moderately reduced plant height and ear height. Together, these results provided new insight into the function of *ZmPHYCs* and guidance for breeding high‐density tolerant maize cultivars.

## Introduction

Maize (*Zea mays* ssp. *mays*) is the world’s highest producing crop now and widely cultivated across all major continents, with an annual planting area of ~197 185 936 hectares (FAO statistics, http://www.fao.org/faostat/zh/#data/QC). Over the past few decades, increasing planting density has made an important contribution to the constant yield increases in the United States, China and other countries (Lee and Tollenaar, 2007; Mansfield and Mumm, 2014; Tollenaar and Lee, 2002). However, shade‐avoidance syndrome (SAS), which is triggered when plants sense a reduction of red‐to‐far‐red light ratios (R:FR < 1.0) in the dense canopy, is detrimental to maize yield production due to increased plant height (and ear height, and less lodging‐resistant), prolonged anthesis–silking interval (ASI), deteriorated tassel development and reduced carbohydrate transport to grains (Boccalandro *et al*., 2009; Dubois and Brutnell, 2011; Duvick, 2005a; Duvick, 2005b; Gonzalo *et al*., 2010; Ku *et al*., 2015). Therefore, dissecting the molecular mechanisms of SAS in maize could provide useful guidance for breeding of high‐density‐tolerant maize cultivars.

Previous studies in Arabidopsis and other plant species have shown that phytochromes (phys), the red/far‐red light photoreceptors, are responsible for perceiving the decline of red‐to‐far‐red light ratios (R:FR) under canopy shade conditions and subsequent triggering of SAS. Arabidopsis has five phy members, phyA‐phyE. Among them, phyA belongs to type I (light labile) phy, and phyB to phyE are type II (light stable) phys (Li *et al*., 2011). phyA is the primary photoreceptor for far‐red light and is responsible for mediating far‐red high‐irradiation response (FR‐HIR) and the very‐low‐fluence response (VLFR), and phyB‐phyE are the photoreceptors for red light‐mediated low‐fluence response (LFR), with phyB playing a major role (Franklin and Quail, 2010; Wang and Wang, 2015).

Phys exist in two forms: the red‐absorbing inactive Pr form and the far‐red‐absorbing biologically active Pfr form (Rockwell *et al*., 2006). The Pr and Pfr conformers can be converted to each other depends on the R:FR ratio. Under high R:FR, red light induces a Pr‐to‐Pfr conformational shift that promotes the translocation of Pfr‐phys into the nucleus to suppress SAS. Under low R:FR, FR converts the Pfr form of phys to the Pr form, and then SAS is induced. More recently, numerous studies showed that phys (mainly phyB) act to repress SAS through regulating the degradation or phosphorylation of phytochrome‐interacting factor (PIF) proteins and then downstream transcriptional regulatory networks (Leivar *et al*., 2012; Lorrain *et al*., 2008; Mansfield and Mumm, 2014; Mizuno *et al*., 2015; Xie *et al*., 2017).

Studies with loss‐of‐function *phyC* mutants in Arabidopsis have revealed that in addition to serving as a weak red light sensor in mediating seedling depolarization and cotyledon enlargement, *phyC* is also a flowering repressor under short‐day (SD) conditions (Franklin *et al*., 2003; Monte *et al*., 2003). In rice, *phyC* plays an important role in FR‐mediated inhibition of coleoptile elongation and the repression of flowering under long‐day (LD) conditions (Takano *et al*., 2005). A role for *phyC* in plant architecture development and flowering time has also been reported in other plants (Chen *et al*., 2014; Nishida *et al*., 2013; Saïdou *et al*., 2009; Takano *et al*., 2005).

The maize genome contains a pair of duplicated genes for *PHYA*, *PHYB* and *PHYC*, named *PHYA1*, *PHYA2*, *PHYB1*, *PHYB2*, *PHYC1* and *PHYC2* (Sheehan *et al*., 2004). Limited studies have shown that the maize *elongated mesocotyl1* (*elm1*) mutant (which is defective in the *ZmHY2* gene encoding phytochromobilin synthase) and the maize *phyB1 phyB2* double mutant have elongated internodes and are prone to lodging (Kebrom *et al*., 2010; Kebrom *et al*., 2006; Sheehan *et al*., 2007), whereas overexpression of *ZmPHYA1* causes increased plant and ear height in maize (Yu *et al*., 2018). In addition, phy‐mediated signalling has been shown to be involved in shade suppression of axillary bud outgrowth in maize (Kebrom *et al*., 2010; Kebrom *et al*., 2006; Whipple et al., 2011). However, the roles of *ZmPHYC1* and *ZmPHYC2* in regulating plant development, flowering time and SAS have not been reported yet.

In this study, we investigated the expression patterns and molecular properties of *ZmPHYC1* and *ZmPHYC*2. Heterologous expression in Arabidopsis revealed a conserved function of *ZmPHYCs* in regulating seedling photomorphogenesis, flowering time and SAS. Using CRISPR/Cas9 technology, we generated *zmphyC1 zmphyC2* double knockout mutants and these mutants showed a moderate early‐flowering phenotype under LD conditions. Moreover, we found that overexpression of *ZmPHYC2* caused a moderate reduction in plant height and ear height. Our results suggest that *ZmPHYC1* and *ZmPHYC2* can be used as potential targets for genetic engineering of maize to improve flowering time and plant architecture suitable for high‐density planting.

## Results

### ZmPHYC1 and ZmPHYC2 proteins have the conserved functional regions

**Figure 1:**
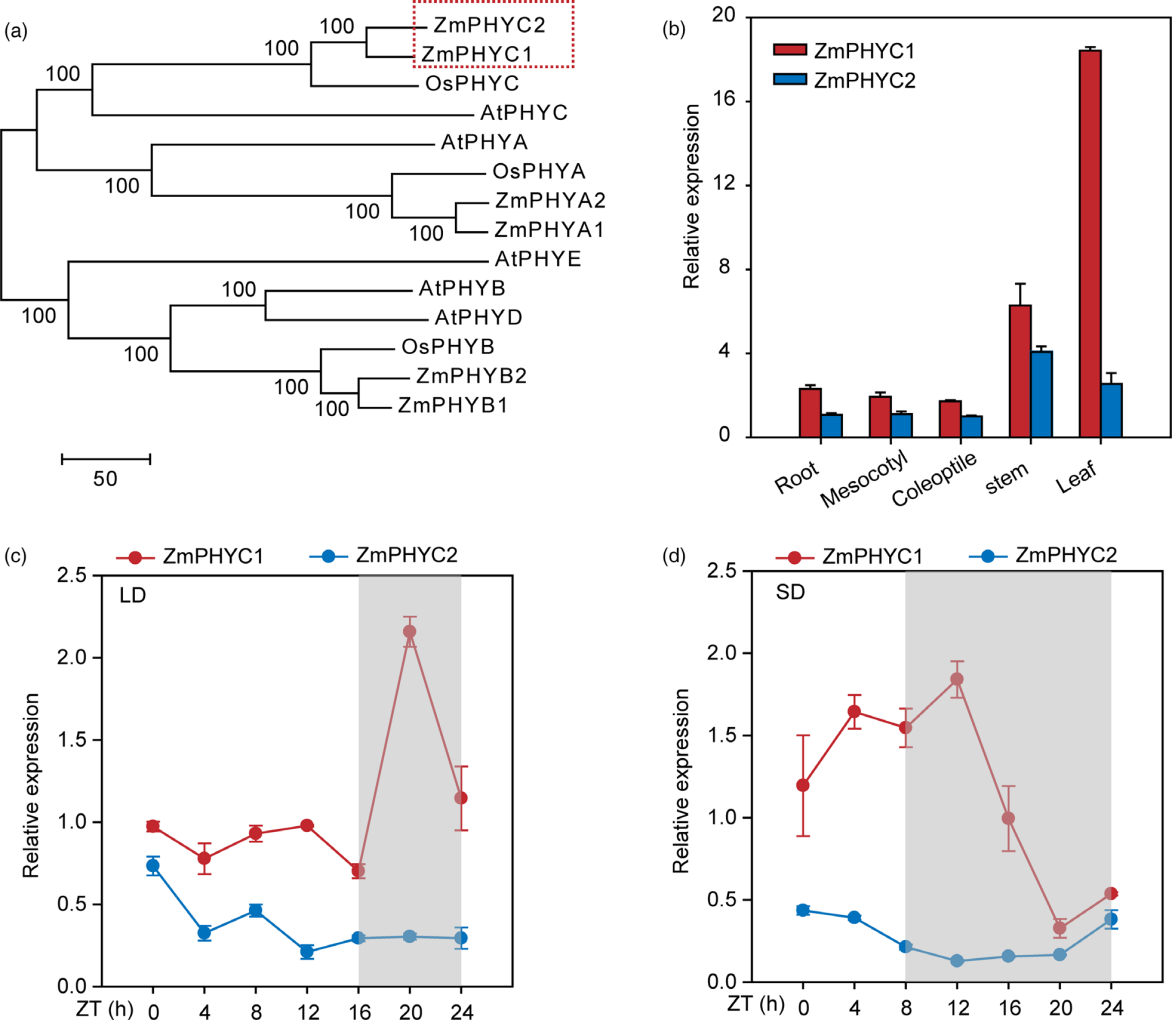
Phylogenetic tree and *ZmPHYC* expression features. (a) Phylogenetic tree of maize phytochrome homologues. At, Os and Zm represent *Arabidopsis thaliana*, *Oryza sativa*, and *Zea mays*, respectively. Full‐length protein sequences of PHYC were obtained from the Gramene database (http://ensembl.gramene.org/genome_browser/index.html) and used to construct the phylogenetic tree with MEGA7 software using the neighbor‐joining method. ZmPHYC1 and ZmPHYC2 are marked with red rectangle. (b) RT‐qPCR analysis shows that *ZmPHYC1* and *ZmPHYC2* are expressed in multiple tissues. Six‐leaf stage seedling of the maize inbred line B73 were used to harvest different tissues for RNA extraction. (c and d) Diurnal expression patterns of *ZmPHYC1* and *ZmPHYC2* in maize inbred line ZC01 leaves under artificial LD (c) and SD (d) conditions. The gray shadows indicate the dark period. The leaves of three‐leaf stage ZC01 seedlings were harvested at different time points under LD or SD conditions. ZT, zeitgeber time. The data are relative to the control gene *Tubulin 5* and represent means ± SD of three biological replicates. LD, long‐day; SD, short‐day.

The two homologs *ZmPHYC* genes [*ZmPHYC1* (Zm00001d034038‐T002) and *ZmPHYC2* (Zm00001d013262‐T004)] were isolated from maize B73 inbred line according to the corresponding annotated sequence. Multiple sequence alignment showed that ZmPHYC1 and ZmPHYC2 proteins have highly conserved domains including a PAS‐2 domain, a cGMP‐specific phosphodiesterases/adenyl cyclase/FhlA (GAF) domain, a phytochrome (PHY) domain, two PAS domains, a His Kinase A (HisKA) domain and a histidine kinase‐like ATPase (HATPase_c) domain (Figure S1). A phylogenetic tree with the sequences of PHYC proteins from Arabidopsis, rice and maize was constructed, and the results indicated that ZmPHYC1 and ZmPHYC2 belong to the type II phy proteins and they share the highest similarity with PHYC of rice (OsPHYC) (Figure 1a).

### Gene expression pattern and subcellular localizations of ZmPHYC1 and ZmPHYC2 proteins

To study the expression profiles of *ZmPHYC* genes in maize plants, tissues from different organs of B73 were collected from plants at the six‐leaf stage grown in a growth room. The results showed that both *ZmPHYC* genes were expressed in all organs examined and that *ZmPHYC1* was expressed at higher levels than *ZmPHYC2* in all organs (Figure 1b), which was in line with the transcriptomic data of maize B73 (Figure S2). The diurnal expression patterns of *ZmPHYCs* were also monitored under artificial LD and SD conditions (Figure 1c and d). Under both LD and SD conditions, the expression of *ZmPHYC1*, but not *ZmPHYC2,* showed an obvious diurnal oscillation pattern. Expression of *ZmPHYC1* peaked at 4 h after dusk (20 h at zeitgeber time) under LD conditions or at the 4 h after dusk under SD conditions (Figure 1c). The distinct temporal expression patterns of the *ZmPHYC1* and *ZmPHYC2* suggest that subfunctionalization may have occurred between them.

To elucidate the localization pattern of ZmPHYCs in cells, we construct a ZmPHYCs‐GFP fusion protein constructs, which were then transiently expressed by infiltration in *N. benthamiana* leaves. As shown in Figure S3, the florescent signals of ZmPHYC1‐GFP and ZmPHYC2‐GFP fusion proteins were observed both in the nucleus and cytoplasm under light conditions.

### ZmPHYC1 and ZmPHYC2 proteins can interact with themselves and ZmPHYBs

Previous studies have reported that phyC is predominantly exists as heterodimers or homodimer in plant cells for function (Chen *et al*., 2014; Monte *et al*., 2003; Takano *et al*., 2005). Thus, we examined the interactions of ZmPHYCs with themselves or with other phy proteins using LCI and BiFC assays in *N. benthamiana* leaf cells. As expected, LCI assays showed that ZmPHYCs could interact with other maize type II phy proteins, ZmPHYB1 and ZmPHYB2 (Figure S4a). Interestingly, ZmPHYC1 and ZmPHYC2 proteins could also interact with themselves and their homologues (Figure S4b). BiFC assays also verified that ZmPHYC proteins could interact with themselves and with ZmPHYB1 and ZmPHYB2 in the nucleus of *N. benthamiana* leaf epidermal cells (Figure S4c). No visible fluorescence signal was detected in the negative controls (Figure S4d). These observations suggest that the interaction with other phytochrome proteins may be required for ZmPHYCs to perform their functions.

### Overexpression of *ZmPHYC1* and *ZmPHYC2* rescues the phenotype of Arabidopsis *phyC‐2* mutants

**Figure 2:**
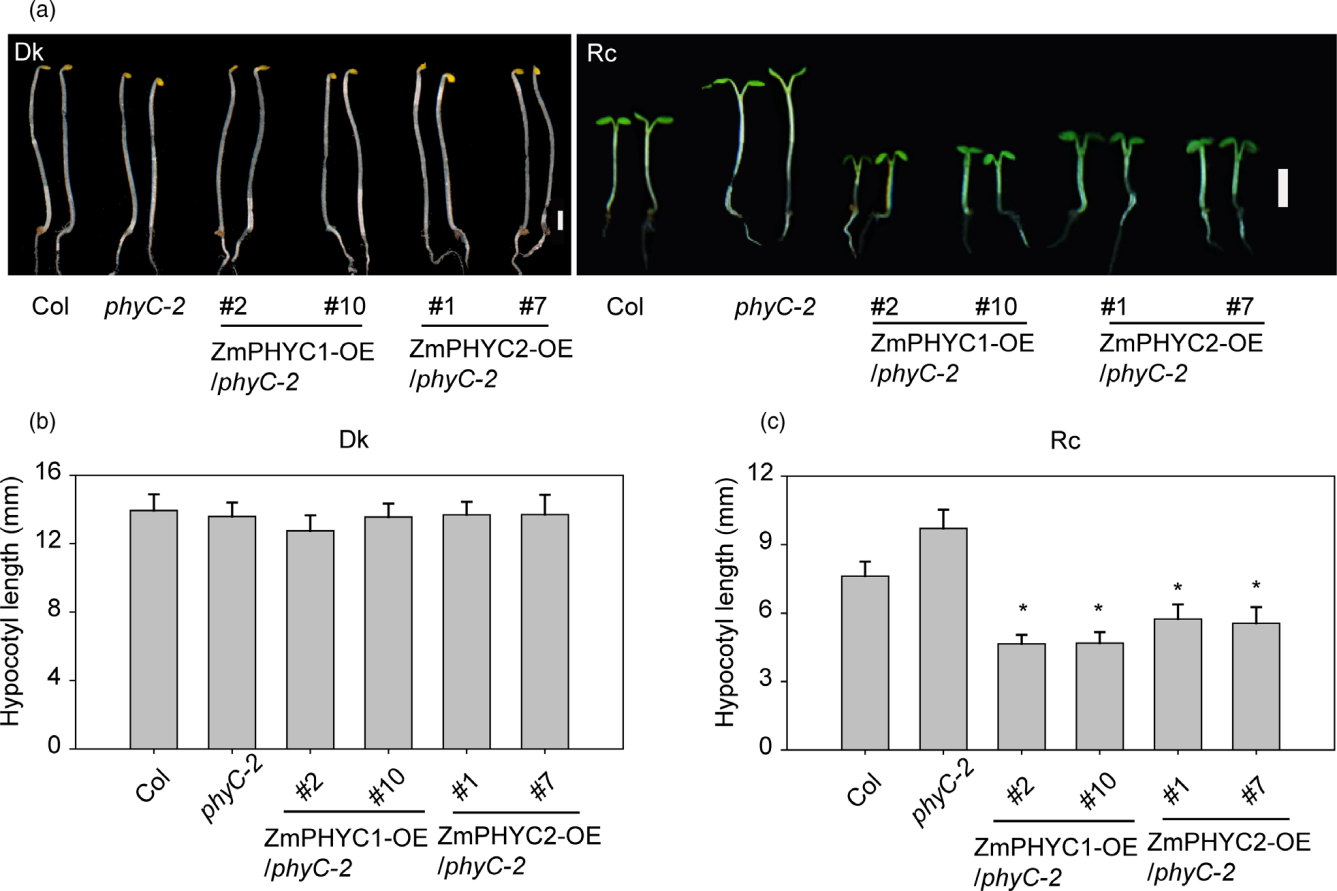
Heterologous expression of *ZmPHYC1* and *ZmPHYC2* rescues the elongated hypocotyl phenotype of the Arabidopsis *phyC‐2* mutant under constant red light (Rc) conditions. (a) Representative 4‐d‐old seedlings of *ZmPHYC1‐OE* and *ZmPHYC2‐OE* transgenic lines in the *phyC‐2* mutant background either grown in the darkness (Dk) or under Rc (30 μmol photons m^−2^ s^−1^). Bars = 2 mm. (b and c) Quantification of hypocotyl lengths of the seedlings shown in (a). Data represent the mean and SD of at least 20 seedlings. Statistical significance analyses were performed between the transgenic plants and *phyC‐2* mutant plants according to student’s *t*‐test. ∗ *P *< 0.01.

The Arabidopsis *phyC‐2* mutant displays a long‐hypocotyl phenotype under continuous red light conditions (Franklin *et al*., 2003; Monte *et al*., 2003). To examine whether *ZmPHYC1* and *ZmPHYC2* could have *PHYC* functions, we tested whether they could complement the *phyC‐2* phenotype by generating *ZmPHYC1‐OE* and *ZmPHYC2‐OE* plants in the Arabidopsis *phyC‐2* mutant background (Figure S5a and b). In darkness, all of the *ZmPHYCs‐OE* lines had normal seedling development and exhibited similar hypocotyl lengths without significant differences (Figure 2a and b). When *ZmPHYC1* and *ZmPHYC2* were overexpressed in the *phyC‐2* background, their hypocotyls were significantly reduced relative to *phyC‐2* under continuous red light (Figure 2a and c). The result suggests that both *ZmPHYC1* and *ZmPHYC2* have conserved function as *AtPHYC*.

We also tested the effect of *ZmPHYCs* on flowering in the Arabidopsis *phyC‐2* mutant background. Unexpectedly, the *ZmPHYC1*‐ and *ZmPHYC2*‐overexpression Arabidopsis plants flowered earlier than *phyC‐2* in LD photoperiods (Figure S6), indicating a promoting role of *ZmPHYC1* and *ZmPHYC2* in the regulation of flowering time.

### *ZmPHYC1* and *ZmPHYC2* are involved in the control of seedlings de‐etiolation under continuous red (Rc) and continuous blue (Bc) light conditions

**Figure 3:**
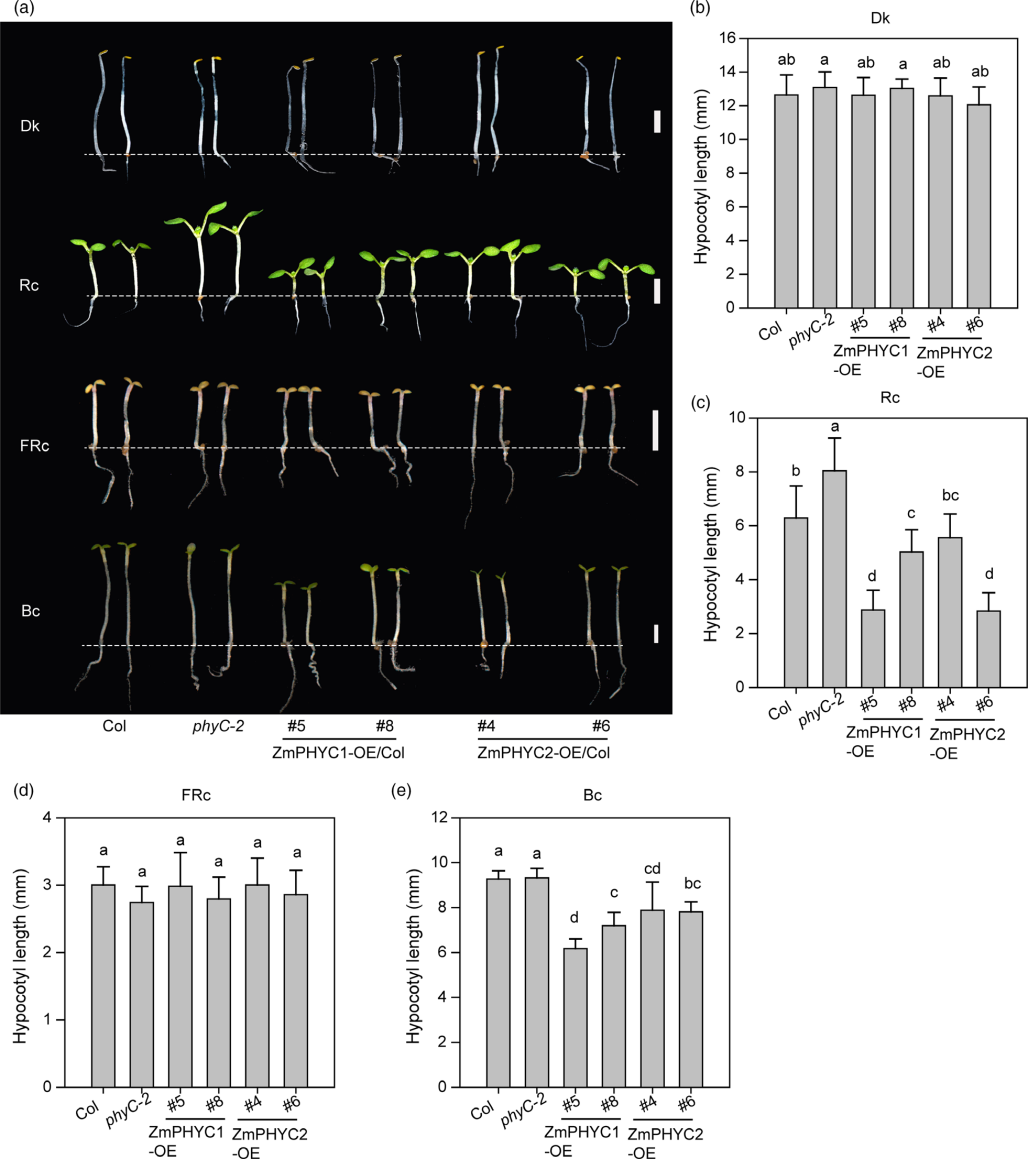
Overexpression of *ZmPHYC1* and*ZmPHYC2* confers a hypersensitive response to red and blue light, but not far‐red light. (a) Phenotype of Col‐0 (wild type), *phyC‐2* mutant, *ZmPHYC1‐OE* and *ZmPHYC2‐OE* transgenic lines (in the wild‐type Col‐0 background) grown in continuous darkness (Dk), red (Rc, 30 μmol photons m^−2^ s^−1^), far‐red (FRc, 2.1 μmol photons m^−2^ s^−1^) or blue (Bc, 1.0 μmol photons m^−2^ s^−1^) light conditions for 4 days. Bars = 2 mm. (b‐e) Quantification of hypocotyl length of the seedling plants shown in (a). Data represent the mean and SD of at least 20 seedlings. Different letters indicate significant differences according to Bonferroni corrected (*P *< 0.05).

To investigate the effect and the possible roles of *ZmPHYCs* in regulating seedling photomorphogenesis, we also overexpressed *ZmPHYCs* (*ProUbi::ZmPHYCs‐GFP*) in the Arabidopsis wild‐type Col‐0 background. Two independent overexpression lines for *ZmPHYC1* (*OE5* and *OE8*) and two independent overexpression lines *ZmPHYC2* (*OE4* and *OE6*) were selected and used to perform further analyses (Figure S5c and d). All the seedlings have no apparent differences in the dark (Figure 3a and b). However, the hypocotyls of all the transgenic lines were significantly shorter than those of Col‐0 in Rc and Bc conditions at the indicated light intensities, which indicates that both *ZmPHYC1* and *ZmPHYC2* can participate in inhibiting hypocotyl elongation growth in Arabidopsis under Rc and Bc (Figure 3a, c, e). Under FRc, *ZmPHYCs‐OE* seedlings failed to suppress the hypocotyl elongation (Figure 3d), indicating that *ZmPHYCs* do not play a major role in the control of hypocotyl elongation in FRc.

Further, we checked the fluence‐rate response of all the *ZmPHYC1‐OE/Col* and *ZmPHYC2‐OE/Col* transgenic lines under Rc, FRc and Bc conditions (Figure S7). Comparative fluence‐response curves for hypocotyl growth indicated that both *ZmPHYC1‐OE* and *ZmPHYC2‐OE* transgenic lines were hypersensitive to Rc from low‐fluence rates (1.6 μmol/m^2^/s) to high‐fluence rates (34 μmol/m^2^/s) tested (Figure S7, left panel). The growth suppression of hypocotyl length was only detected at low‐fluence rate (1.0 and 1.4 μmol/m^2^/s) of Bc (Figure S7, middle panel). However, there was no significant difference for the hypocotyl lengths in different genotype plants under different fluence rates of FRc (Figure S7, right panel). Taken together, these results demonstrate that *ZmPHYCs* are mainly involved in red and blue light‐mediated seedling photomorphogenesis.

### Overexpression of *ZmPHYC1* and *ZmPHYC2* attenuates responses to EOD‐FR treatment in Arabidopsis

**Figure 4:**
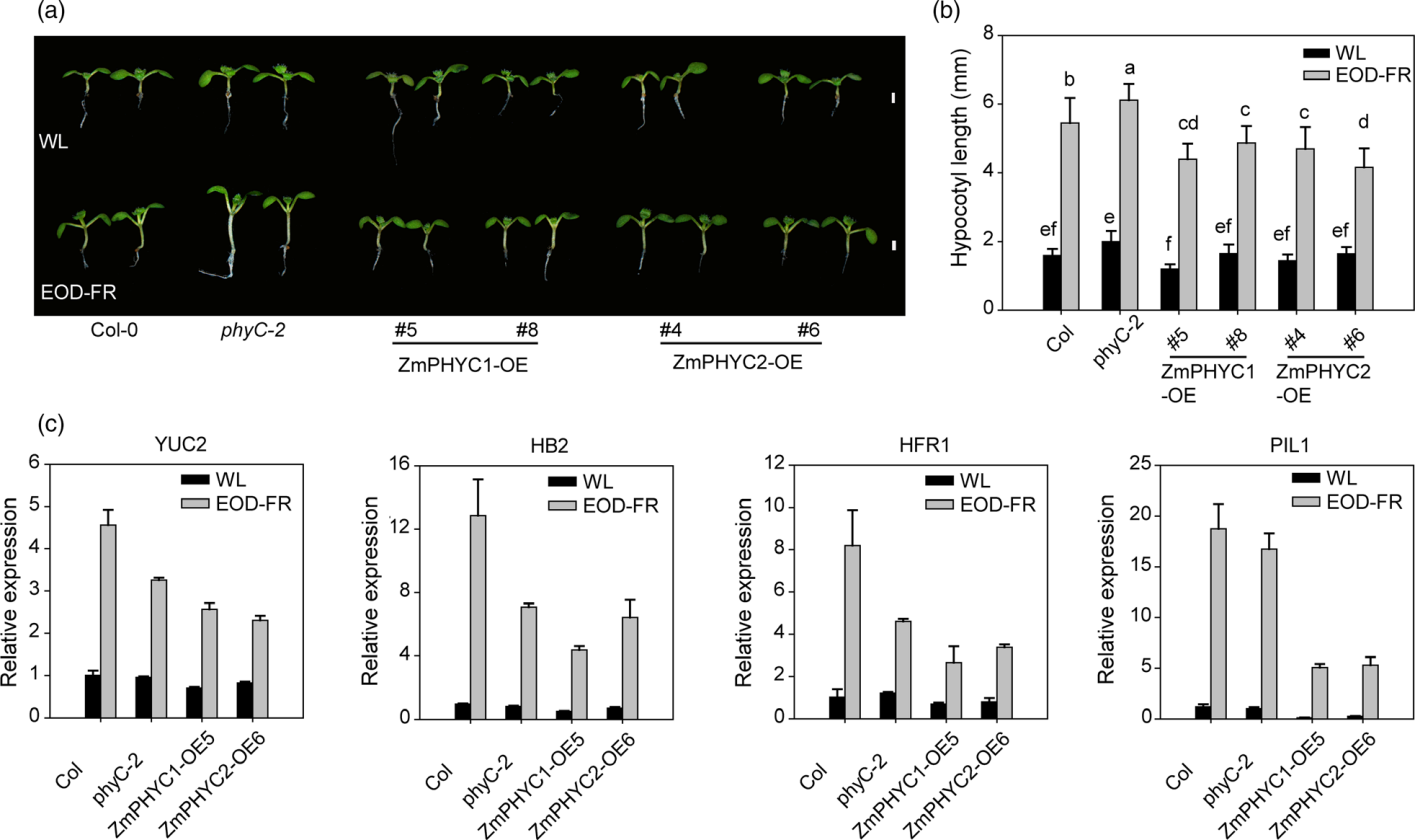
Overexpression of *ZmPHYC1* and *ZmPHYC2* confers an attenuated response to simulated shade treatment in Arabidopsis. (a) Phenotypes of Arabidopsis seedlings grew under normal white light (WL, 55.55 μmol photons m^−2^ s^−1^) or subjected to EOD‐FR treatment. Seedlings of Col‐0 wild type, *phyC‐2*, *ZmPHYC1‐OE* and *ZmPHYC2‐OE* (in the Col‐0 background) were grown under WL conditions for 2 d, and then were kept in WL or treated with FR light (30 μmol m^−2^ s^−1^) for 30 min at the end of the light period (EOD‐FR treatment) for 5 d before photographing. (b) Quantification of hypocotyl lengths for Col‐0, *phyC‐2* mutant, and *ZmPHYC1* or *ZmPHYC2* transgenic lines grown as in (a). Data represent the mean and SD from at least 20 seedlings. Different letters indicate significant differences (*P* < 0.05) according to Bonferroni corrected. Bars = 2 mm. (c) Overexpression of *ZmPHYC1* and *ZmPHYC2* reduces the expression levels of shade response related genes in Arabidopsis. The expression of marker genes including *HFR1*, *YUC2*, *HB2* and *PIL1* was analyzed by RT‐qPCR in 4‐d‐old seedlings grown under WL or subjected to EOD‐FR treatment. *ACT2* was used as the internal control. Data are means and SD of three independent biological replicates.

We next tested whether overexpression of *ZmPHYCs* could alleviate SAS in Arabidopsis. Seedlings of Col‐0, *phyC‐2* and *ZmPHYCs‐OE*/*Col‐0* plants were either grew under normal white light (WL) conditions or a simulated shade condition, in which the seedlings were given an end‐of‐day FR light treatment (EOD‐FR). When grown under normal WL conditions (high R:FR), the hypocotyl lengths had no significant difference between the transgenic lines and the wild‐type plants. While under EOD‐FR treatment, the *ZmPHYC1* and *ZmPHYC2* overexpression lines exhibited obviously shorter hypocotyls compared with the wild‐type plants (Figure 4a, b), indicating an attenuated shade‐avoidance response. RT‐qPCR analysis confirmed that the expression levels of four genes, *LONG HYPOCOTYL IN FAR‐RED1* (*HFR1*), *YUCCA2* (*YUC2*)*, ATHB*‐2 (*HB‐2*) and *PIF3‐LIKE1* (*PIL1*), known to be shade‐induced marker genes (Li *et al*., 2012; Zhang *et al*., 2013), were lower in the *ZmPHYC1* or *ZmPHYC2* overexpression lines than Col‐0 after EOD‐FR treatment (Figure 4c). Altogether, these results suggest that the *ZmPHYCs* act to repress SAS in Arabidopsis.

### *zmphyC1 zmphyC2* double knockout mutants display an early‐flowering phenotype under LD conditions

**Figure 5:**
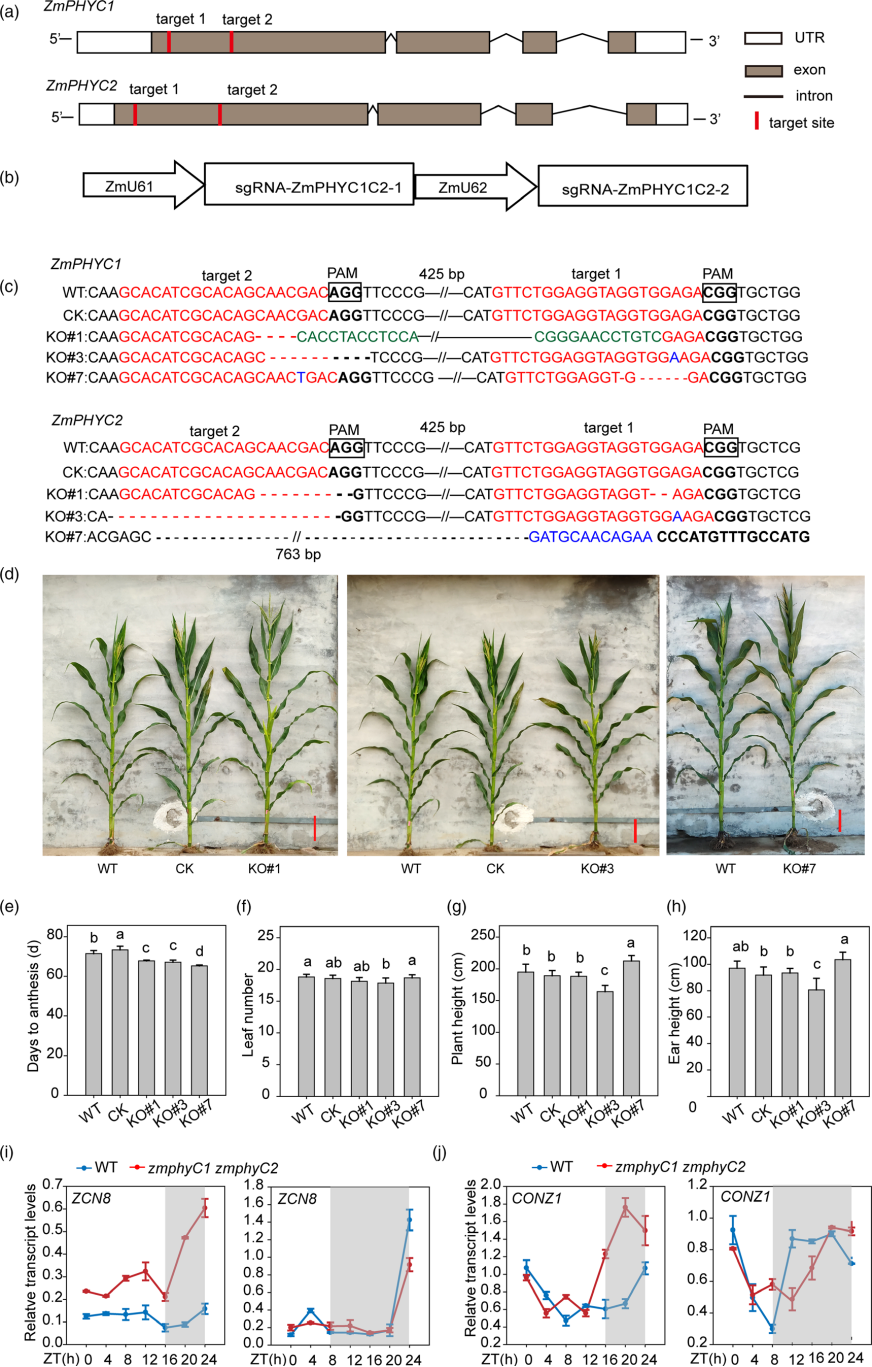
The *zmphyC1 zmphyC2* double knockout mutants display a modest early‐flowering phenotype under natural LD conditions. (a) Diagram showing the two target sites on the *ZmPHYC1* and *ZmPHYC2* genes, respectively. (b) Diagram illustrating the sgRNA expression cassettes targeting *ZmPHYC1* and *ZmPHYC2* genes via the dual‐sgRNAs CRISPR/Cas9 vector system. (c) Sequence analysis of the target sites in three homozygous*zmphyC1 zmphyC2* double knockout lines. The wild type (WT) sequence is shown at the top. The target sites and protospacer‐adjacent motif (PAM) sequences are shown in the antisense strand and highlighted in red and boldface fonts, respectively. Red and black short dashed lines indicate deletions. Inversions and insertions are indicated by green and blue fonts, respectively. The sequence gap length is shown above or under the sequences. (d) Gross morphologies of WT, CK (null segregates with wild‐type *ZmPHYC1* and *ZmPHYC2* genes) and *zmphyC1 zmphyC2* double knockout plants at flowering (anthesis) under natural LD conditions. Bars = 20 cm. (e‐h) Days to anthesis (e), leaf number (f), plant height (g), and ear height (h) in WT, CK and three knockout lines under natural LD conditions. Data represent the mean and SD from at least 10 maize plants. Different letters indicate significant differences (*P* < 0.05) according to Bonferroni corrected. (i and j) Transcript profiles of *ZCN8* (i) and *CONZ1* (j) in the WT (blue lines) and double *zmphyC1 zmphyC2* mutant (red lines) plants. The data are relative to the control gene *Tubulin 5* and represent means ± SD of three biological replicates. Plants were grown under artificial LD and SD conditions. The gray shadows indicate the dark period. ZT, zeitgeber time.

In order to further investigate the function of *ZmPHYCs* in maize growth and development, we generated *zmphyC1 zmphyC2* double knockout mutants in the maize inbred line ZC01 background using the CRISPR/Cas9 technology (Figure 5a, b). Three homozygous lines, named KO*#*1, KO*#*3 and KO*#*7, were identified in the T_1_ generation and selfed for two additional generations to produce T_3_ seeds (Figure 5c). T_3_ homozygous plants without CRISPR vector were planted in natural LD and SD conditions for phenotype observation and statistical analysis. Phenotypic investigation results showed that all the three *zmphyC1 zmphyC2* double mutant lines flowered (anthesis) earlier than wild‐type and transformation control plants (CK, null segregates with wild‐type *ZmPHYC1* and *ZmPHYC2* genes) for 4 to 6 days under LD conditions (Figure 5d and e), without significant differences in the total leaf number between the mutant lines (except KO#3), CK (null segregate) and WT plants (Figure 5f). There were no obvious differences in plant height and ear height between the *zmphyC1 zmphyC2* mutant lines compared with WT and CK plants, except for KO#3, which had a slight shorter plant height and ear height (Figure 5g and h). It was worth mentioning that the *zmphyC1 zmphyC2* double mutants did not show an early‐flowering phenotype under SD conditions (Figure S8). These results suggest that *ZmPHYCs* mainly play a repressive role in flowering under LD conditions.

To understand how *ZmPHYCs* affect flowering at a molecular level, we examined the expression patterns of several known photoperiod and circadian clock genes, including *ZCN8*, *CONZ1*, *ZmCCT9* and *ZmCCA1* (Huang *et al*., 2018; Lazakis *et al*., 2011; Meng *et al*., 2011; Miller *et al*., 2008; Yang *et al*., 2013). Seven‐leaf stage of WT and *zmphyC1 zmphyC2* mutants grew under artificial LD and SD conditions were used for RNA extraction and gene expression analysis. As shown in Figure 5i, *ZCN8*, which encodes the maize florigen and functions as a floral activator (Lazakis *et al*., 2011; Meng *et al*., 2011), exhibited increased expression in the *zmphyC1 zmphyC2* mutants compared with WT plants for all the sampling points during LD conditions, which is in line with their phenotypes. Next, we examined the expression levels of *CONZ1*, a key factor of the photoperiod pathway in maize (Miller *et al*., 2008). The *CONZ1* waveform in the *zmphyC1 zmphyC2* mutant correlated well with that of *ZCN8* under LD conditions, with peak expression at midnight (20 h) (Figure 5j). While under SD conditions, the expression of *CONZ1* was nearly the same between the *zmphyC1 zmphyC2* mutant and WT (Figure 5j). Interestingly, there was no significant difference in the expression of *ZmCCT9* and *ZmCCA1* between WT and the *zmphyC1 zmphyC2* mutant plants (Figure S9), suggesting that the expression of *ZmCCT9* and *ZmCCA1* is not regulated by *ZmPHYCs*.

### Overexpression of *ZmPHYC2*, but not *ZmPHYC1*, modestly reduces maize plant height

**Figure 6:**
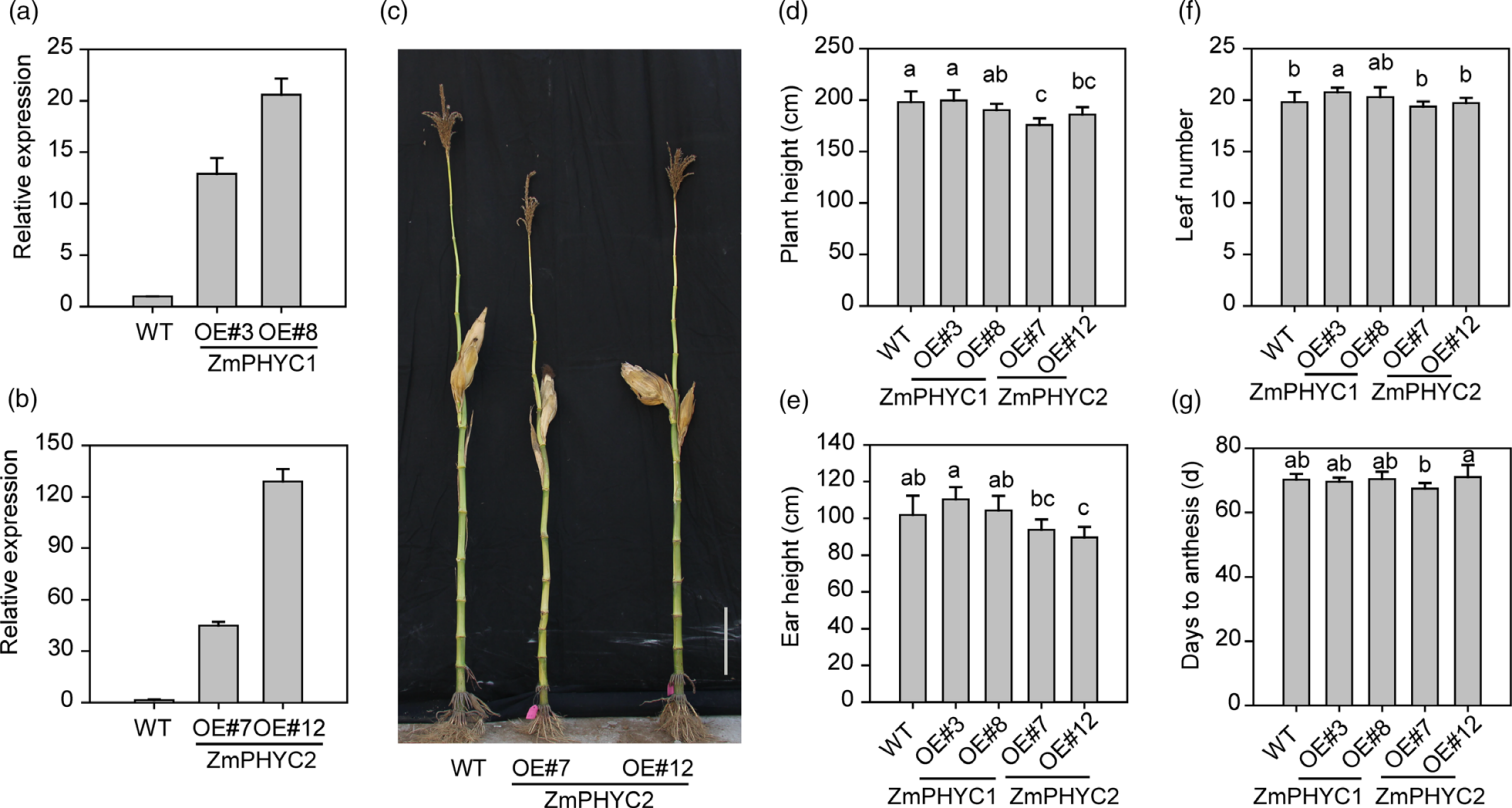
Overexpression of *ZmPHYC2* reduces plant height and ear height in maize. (a and b) RT‐qPCR analysis show that *ZmPHYC1* and *ZmPHYC2* are highly expressed in the maize *ZmPHYCs* overexpression plants. Three‐leaf stage maize seedlings of wild type (WT), *ZmPHYC1‐OE* and *ZmPHYC2‐OE* transgenic plants were used to perform RT‐qPCR analysis. The mRNA level of *Tublin5* was used as a reference. Data are means and SD of three independent biological replicates. (c) The plant height of WT and the transgenic plants overexpressing *ZmPHYC2* in maize under natural long‐day (LD) conditions. Bar = 20 cm. (d‐g) Comparison of plant height (d), ear height (e), leaf number (f), and days to anthesis (g) between WT, *ZmPHYC1‐OE* and *ZmPHYC2‐OE* lines under natural LD conditions. Data represent the mean and SD from at least 10 maize plants. Different letters indicate significant differences (*P* < 0.05) according to Bonferroni corrected.

We also generated *ZmPHYC1* and *ZmPHYC2* overexpression lines in the maize inbred line ZC01. Two independent transgenic maize plants (*OE3* and *OE8* for *ZmPHYC1*, *OE7* and *OE12* for *ZmPHYC2*) with high expression levels of *ZmPHYCs* (Figure 6a, b) were selected and used for subsequent analyses. We observed that the plant height and ear height of the *ZmPHYC2‐OE* lines, but not that of the *ZmPHYC1‐OE* plants, were modestly reduced compared to WT plants, although they had similar total leaf numbers compared with WT (Figure 6c‐f). No significant differences in flowering time were observed in these overexpression lines under LD conditions, compared with the WT plants (Figure 6g).

## Discussion

### ZmphyCs function as type II phytochromes in regulating seedling photomorphogenesis and SAS

In this study, we showed that heterologous expression of both *ZmPHYC1* and *ZmPHYC2* complemented the phenotype of Arabidopsis *phyC‐2* mutant under constant red light conditions and that overexpression of *ZmPHYC1* and *ZmPHYC2* in Arabidopsis (wild‐type background) conferred a hypersensitive response (shorter hypocotyls) to red and blue light irradiation, but not to constant far‐red light (Figures 2 and 3), indicating that ZmphyC1 and ZmphyC2 indeed function as type II phytochromes, as the Arabidopsis and rice phyCs, which have been shown to play a minor role in early seedling development and shade‐avoidance responses in Arabidopsis and rice, respectively (Franklin *et al*., 2003; Takano *et al*., 2005). In addition, we showed that overexpression of *ZmPHYCs* in Arabidopsis significantly reduced the hypocotyl elongation under EOD‐FR treatment (Figure 4), indicating that *ZmPHYCs* also participate in SAS regulation in Arabidopsis. Together, these results suggest that both of ZmphyC1 and ZmphyC2 function as type II phytochormes and act similarly as AtphyC in mediating seedling photomorphogenesis and SAS. Consistent with this notion, we found that ZmPHYCs could form homodimers with themselves or with their homologs as well as form heterodimers with ZmPHYBs (Figure S4), similar to wheat PHYC (Chen *et al*., 2014). However, it was shown that Arabidopsis phyC could not bind to itself in vivo and it only forms heterodimers with phyB (Clack *et al*., 2009; Sharrock and Clack, 2004). Thus, whether homodimerization is a general feature for monocots and dicots PHYCs remains to be further investigated.

It is notable that we showed that although both *ZmPHYC1* and *ZmPHYC2* are actively expressed in all tissues examined, *ZmPHYC1* has significantly higher expression levels than *ZmPHYC2* (Figure 1b, S2). In addition, it appears that the expression of *ZmPHYC1*, but not *ZmPHYC2*, is subject to regulation by the circadian clock (Figure 1c and d). These results suggest that subfunctionalization may have occurred between them. Consistent with this, we found that overexpression of *ZmPHYC2*, but not *ZmPHYC1*, causes a modest reduction in the plant height and ear height in the transgenic maize plants (Figure 6). Future studies are required to elucidate the functional differentiation of *ZmPHYC1* and *ZmPHYC2* in regulating different developmental and physiological processes.

### *ZmPHYCs* as potential targets for fine‐tuning plant height of maize cultivars for adapting to high‐density planting

Previous studies have shown that high‐density tolerant maize inbred and hybrids lines are characterized with several important morphological changes in plant architecture, including reduced ear height and increased culm strength for lodging resistance (Dubois and Brutnell, 2011; Gonzalo *et al*., 2010; Ku *et al*., 2015; Mansfield and Mumm, 2014). It is well known now that SAS causes exaggerated elongated growth, and the stalk lodging caused by high‐density planting is still a major challenge in maize production. The plant height and ear height of maize are controlled by many minor effective QTLs; thus, it is difficult and inefficient to reduce the plant height/ear height by pyramiding these QTLs through conventional maize breeding. In rice and wheat, the utilization of semi‐dwarf mutants *sd1* and *rht1* in the 1960s directly fostered the ‘First Green Revolution’ in crop breeding (Khush, 2001; Rutger and Mackill, 2001; Spielmeyer *et al*., 2002). However, in maize, some extreme dwarf mutants (such as *d3, br2* etc.) could not be directly utilized in maize breeding due to strong negative effects (Multani *et al*., 2003; Winkler and Helentjaris, 1995). Therefore, elite semi‐dwarf maize breeding materials are still rare and highly desirable for breeding of high‐density tolerant maize cultivars that confer lodging resistant with minimal penalty on harvest index. In this study, we showed that overexpression of *ZmPHYC2* conferred a modestly reduced plant height (and ear height), which may represent an ideal trait that can be introgressed into maize breeding lines to increase lodging resistance.

### *ZmPHYCs* are of value in fine‐tuning flowering time in breeding maize cultivars for adapting to local environments

Flowering time is another important agronomic trait, which determines the regional adaptation and yield of crop cultivars. Maize was domesticated in southwestern Mexico from its progenitor teosinte (Matsuoka *et al*., 2002), which has strong photoperiod sensitivity, requiring SD conditions to flower (Hung *et al*., 2012), whereas modern maize is often characterized as day‐neutral plant and cultivated all over the world within a broad range of latitudes. The widespread latitudinal expansion of modern maize benefits from the relaxation of photoperiod sensitivity for flowering for adapting to LD environments and different latitudes (Camus‐Kulandaivelu *et al*., 2006; Chardon *et al*., 2004). Phytochromes are known to play a major role in sensing the photoperiod and flowering time control in a wide range of plant species (Childs *et al*., 1995; Halliday *et al*., 1994; Hanumappa *et al*., 1999; Reed *et al*., 1993; Sawers *et al*., 2002; Sheehan *et al*., 2007). Recently, a role of *PHYC* in regulating flowering time has been well established in a number of plant species, including Arabidopsis, rice, wheat, barley, *Brachypodium distachyon* and pearl millet (Balasubramanian *et al*., 2006; Chen *et al*., 2014; Nishida *et al*., 2013; Saïdou *et al*., 2009; Takano *et al*., 2005; Woods *et al*., 2014). We showed in this study that the *zmphyC1 zmphyC2* double knockout mutants exhibited a moderate early‐flowering phenotype under LD photoperiod (Figure 5), suggesting that *ZmPHYCs* function as a repressor in maize flowering under LD photoperiod. This result is consisting with the reported roles of *PHYC* in Arabidopsis and rice under noninductive photoperiod conditions [i.e. short days for Arabidopsis and long days for rice] (Monte *et al*., 2003; Takano *et al*., 2005). Surprisingly, we observed that overexpression of *ZmPHYCs* promoted flowering in the Arabidopsis *phyC‐2* mutant background in LD conditions (Figure S6). We speculated that the differential roles of *ZmPHYCs* in regulating flowering time in maize and Arabidopsis are somewhat related to maize as an original SD plant, while Arabidopsis is classified as a typical LD plant. Consistent with this notion, loss‐of‐function mutations for *PHYC* in wheat have strong delayed flowering under LD photoperiod, indicating that *PHYC* functions as a flowering accelerator under the inductive photoperiod (Chen *et al*., 2014).

Despite being an important agronomic trait, the genetic control of flowering in maize is still not well studied. Although a large number of quantitative trait locus (QTLs) for flowering time have been mapped in maize, only *Vegetative to generative transition 1* (*Vgt1*) and *ZmCCTs* have been well characterized and found to contribute to flowering‐time adaptation (Huang *et al*., 2018; Hung *et al*., 2012; Salvi *et al*., 2007; Yang *et al*., 2013). The best known gene *ZmCCT10*, encoding a CCT domain‐containing protein, is a homologue of the rice photoperiod response regulator *Ghd7* (Hung *et al*., 2012). The insertion of a Harbinger‐like transposable element (TE) in the promoter of *ZmCCT9* and a CACTA‐like TE in the promoter of *ZmCCT10* dramatically reduced flowering time under LD environment, and these insertions are thought to be selected during spread of maize from its tropical origin to higher latitudes (Huang *et al*., 2018; Yang *et al*., 2013). Natural variations of *PHYCs* have been shown to be correlated with flowering time in Arabidopsis, wheat and pearl millet (Balasubramanian *et al*., 2006; Chen *et al*., 2014; Saïdou *et al*., 2009); thus, it will be interesting to examine whether *ZmPHYCs* are targets of domestication and genetic improvement for adapting to different local environments in the future. In this study, we showed that the *zmphyc1 zmphyC2* double mutants display a modest early‐flowering phenotype (4–6 days), which represents a desirable feature that can be utilized in maize breeding to expand the geographical zone of maize cultivars with minimal impact on the biomass and harvest index. Thus, *ZmPHYC1* and *ZmPHYC2* could serve as valuable targets of molecular breeding of maize cultivars for adapting to different local environments.

## Materials and methods

### Plant materials and growth conditions

The *Arabidopsis thaliana phyC‐2* mutant used in this study was described by Monte *et al*. (2003), and the wide‐type control plants were ecotype Columbia‐0 (Col‐0). Arabidopsis seeds were sown on one‐half‐strength Murashige and Skoog (MS) solid medium containing 1% sucrose and 0.8% agar after surface sterilization and stratified at 4 °C in the darkness for 3 day. Seeds were then exposed to white light for 6 h to induce germination before further treatments.

*Nicotiana benthamiana* seeds were directly sown into the soil and grew in the growth house under a 16‐h light/8‐h dark cycle at 25 °C for one month before being used for the bimolecular fluorescence complementation (BiFC) and luciferase complementation imaging (LCI) assays.

The wild‐type maize inbred line used in this study was ZC01, a private receptor inbred line conducted by the China National Seed Group Co., LTD (Wuhan, China). All the mutant and transgenic lines of maize were in the ZC01 background. For field experiments, ZC01 and *zmphyC* knockout mutants as well as *ZmPHYCs‐OE* transgenic lines were planted in Langfang (39°N, 116°E), Hebei Province, China, during the summer of 2019 and in Ledong (18°N, 116°E), Hainan Province, China, during the winter of 2018. The spacing between rows and plants was set to 30 cm and 15 cm, respectively. Ten plants were selected randomly for scoring of flowering time (days to anthesis, DTA) and several other important agronomic traits including total leaf number, plant height and ear height.

### Total RNA extraction and RT‐qPCR assay

Total RNA was isolated using TRIzol reagent (Invitrogen, USA), and reverse transcription reactions were performed following the manufacturer’s instructions of the FastQuant RT Kit (with gDNase) (Tiangen Biotech, China). Quantitative Real‐time PCR (RT‐qPCR) was performed using the SuperReal PreMix Plus Kit (SYBR Green) (Tiangen Biotech, China) on an Applied Biosystems Q3 real‐time PCR detection system according to the manufacturer’s manual. The transcript levels of *Tubulin 5* (GRMZM2G099167) and *ACT2* (AT3G18780) were used as internal controls for RT‐qPCR in maize and Arabidopsis, respectively. All the primer sequences used for RT‐qPCR above are shown in Table S1.

### Expression profiling of *ZmPHYCs*

For maize tissue expression analysis, B73 seedlings were grown in the growth chamber under long‐day conditions (LD, 16‐h light/8‐h dark) at 28°C. Three weeks after planting, B73 seedlings were harvested and separated into roots, mesocotyls, coleoptiles and leaves for detection of the tissue expression patterns of *ZmPHYCs*. For circadian clock analysis, the leaves of ZC01 seedlings were harvested at different Zeitgeber times at the three‐leaf stage to measure the diurnal expression patterns of *ZmPHYCs*.

### Plasmid construction

To generate constructs for the subcellular localization assay, the coding sequences of *ZmPHYC1* and *ZmPHYC2* without the terminators were amplified from cDNA of the maize inbred line B73 and cloned into the pCambia1305::GFP vector digested with *Xba*I. For transformation of Arabidopsis and maize, we cloned the coding regions of *ZmPHYC1* and *ZmPHYC2* into the CPB‐ProUbi::GFP vector (Zhao *et al*., 2016) through the *Pst*I and *BamH*I sites to generate *ProUbi::ZmPHYC1‐GFP* and *ProUbi::ZmPHYC2‐GFP* constructs.

To generate constructs for the LCI assay, the full‐length coding regions of *ZmPHYB*1, *ZmPHYB2*, *ZmPHYC*1 and *ZmPHYC2* were cloned into the pCAMBIA1300‐nLuc vector digested by the *Sal*I/*Kpn*I sites to generate *ZmPHYB1‐nLuc*, *ZmPHYB2‐nLuc*, *ZmPHYC1‐nLuc* and *ZmPHYC2‐nLuc*, respectively. The full‐length coding regions of *ZmPHYC*1 and *ZmPHYC2* were cloned into the pCAMBIA1300‐cLuc vector digested by the *Sal*I/*Kpn*I sites to produce the corresponding construct *ZmPHYC1‐cLuc* and *ZmPHYC2‐cLuc*, respectively. The vectors for the LCI assay (pCAMBIA1300‐nLuc and pCAMBIA1300‐cLuc) were described previously in Chen *et al*. (2008).

To prepare constructs for the BiFC assay, the full‐length coding sequences of *ZmPHYC*1 and *ZmPHYC2* were recombined into the p2YN vector at the *Pac*I/*Spe*I sites to generate *ZmPHYC1‐p2YN* and *ZmPHYC2‐p2YN*, respectively. The full‐length coding sequences of *ZmPHYB*1, *ZmPHYB2*, *ZmPHYC*1 and *ZmPHYC2* were recombined into the p2YC vector at the *Pac*I/*Spe*I sites to generate *ZmPHYB1‐p2YC*, *ZmPHYB2‐p2YC*, *ZmPHYC1‐p2YC* and *ZmPHYC2‐p2YC*, respectively. The p2YN and p2YC vectors were described previously (Sun *et al*., 2017; Zheng *et al*., 2019).

All the plasmids above were constructed using an In‐Fusion HD Cloning Kit (Clontech) following the manufacturer’s protocol, and all the primers used for the constructs are shown in Table S1.

### Subcellular localization analysis

The *pCambia1305‐35S::ZmPHYC1‐GFP* and *pCambia1305‐35S::ZmPHYC2‐GFP* constructs were introduced into *Agrobacterium tumefaciens* strain EHA105, respectively. *N. benthamiana* leaves were co‐injected with *pCambia1305‐35S::ZmPHYC1‐GFP* or *pCambia1305‐35S::ZmPHYC2‐GFP* together with a nuclear protein marker construct (*Pro‐35S::mRFP‐AHL22*) (Xiao *et al*., 2009). After injection, the *N. benthamiana* plants were incubated in darkness for 12 h and then transferred to greenhouse (16 h light/8 h dark) for 24 to 36 h. GFP and RFP signals were observed using a confocal microscopy (Zeiss LSM710).

### Arabidopsis transformation and phenotypic analysis

The *ProUbi::ZmPHYC1‐GFP* and *ProUbi::ZmPHYC2‐GFP* constructs were transformed into *Agrobacterium tumefaciens* strain GV3101 and further transformed into Arabidopsis wild‐type (Col‐0) and *phyC‐2* mutant using the floral dip method (Clough and Bent, 1998). At least 10 independent lines of each transformation were selected with 0.005% (volume ratio) Basta solution (Coolaber, China) and verified by RT‐qPCR analysis. Two independent transgenic lines of each transgene were selected for further studies.

For phenotypic analysis, Arabidopsis seedlings were moved to constant red light (Rc), or far‐red light (FRc), or blue light (Bc) conditions for 4 day at 22 °C before hypocotyl length measurement. Hypocotyl lengths were measured from the digital photographs using the ImageJ software (version 1.38). The different light intensities were designed as previously described (Franklin *et al*., 2003; Monte *et al*., 2003). For EOD‐FR treatment, 2‐day‐old seedlings were treated with FR light (30 μmol photons/m^2^/s) for 30 min at the end of the light period for 4 day before measurements were taken.

### LCI and BiFC assays

All constructs for LCI and BiFC assays were individually transformed into *Agrobacterium tumefaciens* strain EHA105 and then infiltrated into the leaves of *N. benthamiana* with different combinations as indicated in the figures. For BiFC assay, the *N. benthamiana* leaves were co‐infiltrated with a combination of *Agrobacterium tumefaciens* strain EHA105 carrying the indicated plasmid pairs and the *35S::mRFP‐AHL22* plasmid (Xiao *et al*., 2009). Samples were incubated in darkness for 24 h after the infiltration and then transferred to WL conditions (16‐h light/8‐h dark) for 24 to 36 h. For imaging the luciferase luminescence, the leaves were detached and photographed using the NightShade LB985 Plant Imaging System (Berthold Technologies) with a 60 s exposure time, 4 × 4 binning, slow readout and high gain after spraying with 20 mg/mL potassium luciferin (Gold Biotech, USA). For imaging the fluorescence of the reunion in BiFC, the *N. benthamiana* leaves were observed under confocal microscopy (Zeiss LSM710). All these experiments were independently repeated at least three times.

### Generation and analysis of CRISPR/Cas9 knockout lines of *zmphyC1 zmphyC2*

The CRISPR/Cas9 knockout vector was constructed according to a previously described protocol (Wu *et al*., 2019). Briefly, two targets (target 1 and target 2) were designed in the first exons of *ZmPHYC1* and *ZmPHYC2* genes according to the reported criteria of 5′‐GG‐(N)18‐NGG‐3′ (Figure 5a). Given the *ZmPHYC1* and *ZmPHYC2* genes are highly homologous to each other, two identical target sequences in the exons of these two candidate genes were selected for Cas9 cleavage. The sequences for the guide RNAs, driven by the maize ubiquitin U6‐1 and U6‐2 promoters, respectively (Figure 5b), were inserted into the CPB vector (Zhao *et al*., 2016) using the HindⅢ restriction site and an In‐Fusion HD Cloning Kit (TaKaRa). The primer pair U6‐1‐1F and sgR‐R was used to assemble the first sgRNA expression cassette, and the primer pair U6‐2‐2F and sgR‐R was used to assemble the second sgRNA expression cassette. The primers are shown in Table S1. The resulting vector was confirmed by sequencing and introduced into the recipient line ZC01 via *Agrobacterium tumefaciens*‐mediated transformation. For genotyping, the target sequences of each *ZmPHYC* gene were amplified from ZC01 and the transgenic lines, and then PCR products were directly sequenced. The mutated sequences of each *ZmPHYC* gene in the transgenic lines were revealed by aligning the sequences between the transgenic lines and ZC01. Knockout lines with deletion or frameshift mutations were self‐pollinated to produce homozygous knockout mutants. The primers used above for construction and identifications are shown in Table S1.

### Generation of *ZmPHYC* overexpression transgenic lines in maize

To get the *ZmPHYC* overexpression transgenic lines in maize, the *Agrobacterium tumefaciens* strain EHA105 carrying the *ProUbi::ZmPHYCs‐GFP* construct was used to transform the maize inbred line ZC01 as above described. The positive transgenic individuals of different lines were selected by spraying with the Basta solution (0.2 g/L). Two independent high expression transgenic lines for each *ZmPHYC* gene were verified by RT‐qPCR analysis and selected for further research. The primers used above for identifications are shown in Table S1.

### Statistical analysis

All real‐time PCRs and other quantitative analysis were repeated at least three times. The significant differences of hypocotyl length and flowering time between the *ZmPHYCs‐OE/phyC‐2* transgenic Arabidopsis plants and the *phyC‐2* mutant plants were analysed by Student’s *t*‐test. To evaluate the significant differences of hypocotyl length among the various Arabidopsis genotypes treated or untreated with EOD‐FR and the differences of phenotypes among transgenic maize lines, the multiple comparisons were adopted by the LSD method with R package *agricolae* (R Core Team, 2018). The statistical significance of multiple comparisons was defined by using a Bonferroni‐corrected *P*‐value < 0.05.

## Conflict of interest

The authors declare no conflict of interest.

## Author contributions

H.W. designed the project. Q.L. and G.W. conducted the experiments. Y.Z., B.W. and B.Z. participated in some experiments. Q.L. and G.W. analysed the data and wrote the manuscript. H.W. and C.C. revised the manuscript. All the authors read and approved the manuscript.

## Supporting information


**Figure S1** Multiple sequence alignment of the protein sequences of ZmPHYC1, ZmPHYC2, OsPHYC and AtPHYC.
**Figure S2** A heat map illustrating the expression levels of the *ZmPHYC* genes in different tissues from various developmental stages.
**Figure S3** ZmPHYC1 and ZmPHYC2 proteins are localized in both the nucleus and cytoplasm in *N. benthamiana* epidermal cells under light conditions.
**Figure S4** ZmPHYCs can interact with themselves and ZmPHYBs in plant cells.
**Figure S5** Expression analysis of the *ZmPHYC* transgenes in the selected Arabidopsis transgenic lines.
**Figure S6** *ZmPHYC1‐* and *ZmPHYC2*‐overexpression plants in the *phyC‐2* mutant background shows accelerated flowering compared with the *phyC‐2* mutant.
**Figure S7** Fluence‐rate response curves for hypocotyl length under Rc, Bc and FRc light conditions.
**Figure S8** The *zmphyC1 zmphyC2* double knockout mutants do not show an early‐flowering phenotype under natural SD conditions.
**Figure S9** Transcript profiles of *ZmCCT9* and *ZmCCA1* genes in the wild type (blue lines) and *zmphyC1 zmphyC2* double mutant (red lines).
**Table S1** Primers used in this study.

## References

[pbi13429-bib-0001] Balasubramanian, S. , Sureshkumar, S. , Agrawal, M. , Michael, T.P. , Wessinger, C. , Maloof, J.N. , Clark, R. *et al*. (2006) The PHYTOCHROME C photoreceptor gene mediates natural variation in flowering and growth responses of *Arabidopsis thaliana* . Nat. Genet. 38, 711–715. 16732287 10.1038/ng1818 PMC1592229

[pbi13429-bib-0002] Boccalandro, H.E. , Rugnone, M.L. , Moreno, J.E. , Ploschuk, E.L. , Serna, L. , Yanovsky, M.J. and Casal, J.J. (2009) Phytochrome B enhances photosynthesis at the expense of water‐use efficiency in Arabidopsis. Plant Physiol. 150, 1083–1092. 19363093 10.1104/pp.109.135509 PMC2689964

[pbi13429-bib-0003] Camus‐Kulandaivelu, L. , Veyrieras, J.B. , Madur, D. , Combes, V. , Fourmann, M. , Barraud, S. , Dubreuil, P. *et al*. (2006) Maize adaptation to temperate climate: relationship between population structure and polymorphism in the Dwarf8 gene. Genetics, 172, 2449–2463. 16415370 10.1534/genetics.105.048603 PMC1456379

[pbi13429-bib-0004] Chardon, F. , Virlon, B. , Moreau, L. , Falque, M. , Joets, J. , Decousset, L. , Murigneux, A. *et al*. (2004) Genetic architecture of flowering time in maize as inferred from quantitative trait loci meta‐analysis and synteny conservation with the rice genome. Genetics, 168, 2169–2185. 15611184 10.1534/genetics.104.032375 PMC1448716

[pbi13429-bib-0005] Chen, H. , Zou, Y. , Shang, Y. , Lin, H. , Wang, Y. , Cai, R. , Tang, X. *et al*. (2008) Firefly luciferase complementation imaging assay for protein‐protein interactions in plants. Plant Physiol. 146, 368. 18065554 10.1104/pp.107.111740 PMC2245818

[pbi13429-bib-0006] Chen, A. , Li, C. , Hu, W. , Lau, M.Y. , Lin, H. , Rockwell, N.C. , Martin, S.S. *et al*. (2014) Phytochrome C plays a major role in the acceleration of wheat flowering under long‐day photoperiod. Proc. Natl. Acad. Sci. USA, 111, 10037–10044. 24961368 10.1073/pnas.1409795111 PMC4104863

[pbi13429-bib-0007] Childs, K.L. , Lu, J.L. , Mullet, J.E. and Morgan, P.W. (1995) Genetic regulation of development in sorghum bicolor (X. greatly attenuated photoperiod sensitivity in a phytochrome‐deficient sorghum possessing a biological clock but lacking a red light‐high irradiance response). Plant Physiol. 108, 345–351. 12228479 10.1104/pp.108.1.345 PMC157340

[pbi13429-bib-0008] Clack, T. , Shokry, A. , Moffet, M. , Liu, P. , Faul, M. and Sharrock, R.A. (2009) Obligate heterodimerization of Arabidopsis phytochromes C and E and interaction with the PIF3 basic helix‐loop‐helix transcription factor. Plant Cell, 21, 786–799. 19286967 10.1105/tpc.108.065227 PMC2671712

[pbi13429-bib-0009] Clough, S.J. and Bent, A.F. (1998) Floral dip: a simplified method for *Agrobacterium*‐mediated transformation of *Arabidopsis thaliana* . Plant J. 16, 735–743. 10069079 10.1046/j.1365-313x.1998.00343.x

[pbi13429-bib-0010] Dubois, P.G. and Brutnell, T.P. (2011) Topology of a maize field: distinguishing the influence of end‐of‐day far‐red light and shade avoidance syndrome on plant height. Plant Signal. Behavior, 6, 467–470. 10.4161/psb.6.4.14305 PMC3142371 21364314

[pbi13429-bib-0011] Duvick, D.N. (2005a) The contribution of breeding to yield advances in maize (*Zea mays* L.). Adv. Agronomy, 86, 83–145.

[pbi13429-bib-0012] Duvick, D.N. (2005b) Genetic progress in yield of United States maize (*Zea mays* L.). Maydica, 50, 193–202.

[pbi13429-bib-0013] Franklin, K.A. and Quail, P.H. (2010) Phytochrome functions in Arabidopsis development. J. Exp. Bot. 61, 11–24. 19815685 10.1093/jxb/erp304 PMC2800801

[pbi13429-bib-0014] Franklin, K.A. , Davis, S.J. , Stoddart, W.M. , Vierstra, R.D. and Whitelam, G.C. (2003) Mutant analyses define multiple roles for phytochrome C in Arabidopsis photomorphogenesis. Plant Cell, 15, 1981–1989. 12953105 10.1105/tpc.015164 PMC181325

[pbi13429-bib-0015] Gonzalo, M. , Holland, J.B. , Vyn, T.J. and McIntyre, L.M. (2010) Direct mapping of density response in a population of B73 × Mo17 recombinant inbred lines of maize (*Zea Mays* L.). Heredity, 104, 583–599. 19888291 10.1038/hdy.2009.140

[pbi13429-bib-0016] Halliday, K.J. , Koornneef, M. and Whitelam, G.C. (1994) Phytochrome B and at least one other phytochrome mediate the accelerated flowering response of *Arabidopsis thaliana* L. to low red/far‐red ratio. Plant Physiol. 104, 1311–1315. 12232170 10.1104/pp.104.4.1311 PMC159295

[pbi13429-bib-0017] Hanumappa, M. , Pratt, L.H. , Cordonnier‐Pratt, M.‐M. and Deitzer, G.F. (1999) A photoperiod‐insensitive barley line contains a light‐labile Phytochrome B. Plant Physiol. 119, 1033. 10069841 10.1104/pp.119.3.1033 PMC32084

[pbi13429-bib-0018] Huang, C. , Sun, H. , Xu, D. , Chen, Q. , Liang, Y. , Wang, X. , Xu, G. *et al*. (2018) *ZmCCT9* enhances maize adaptation to higher latitudes. Proc. Natl. Acad. Sci. USA, 115, E334–E341. 29279404 10.1073/pnas.1718058115 PMC5777075

[pbi13429-bib-0019] Hung, H.Y. , Shannon, L.M. , Tian, F. , Bradbury, P.J. , Chen, C. , Flint‐Garcia, S.A. , McMullen, M.D. *et al*. (2012) *ZmCCT* and the genetic basis of day‐length adaptation underlying the postdomestication spread of maize. Proc. Natl. Acad. Sci. USA, 109, E1913–1921. 22711828 10.1073/pnas.1203189109 PMC3396540

[pbi13429-bib-0020] Kebrom, T.H. , Burson, B.L. and Finlayson, S.A. (2006) Phytochrome B represses *Teosinte Branched1* expression and induces sorghum axillary bud outgrowth in response to light signals. Plant Physiol. 140, 1109–1117. 16443694 10.1104/pp.105.074856 PMC1400571

[pbi13429-bib-0021] Kebrom, T.H. , Brutnell, T.P. and Finlayson, S.A. (2010) Suppression of sorghum axillary bud outgrowth by shade, phyB and defoliation signalling pathways. Plant, Cell Environ. 33, 48–58. 19843258 10.1111/j.1365-3040.2009.02050.x

[pbi13429-bib-0022] Khush, G.S. (2001) Green revolution: the way forward. Nat. Rev. Genet. 2, 815–822. 11584298 10.1038/35093585

[pbi13429-bib-0023] Ku, L. , Zhang, L. , Tian, Z. , Guo, S. , Su, H. , Ren, Z. , Wang, Z. *et al*. (2015) Dissection of the genetic architecture underlying the plant density response by mapping plant height‐related traits in maize (*Zea mays* L.). Mol. Genet. Genom. 290, 1223–1233. 10.1007/s00438-014-0987-1 25566854

[pbi13429-bib-0024] Lazakis, C.M. , Coneva, V. and Colasanti, J. (2011) *ZCN8* encodes a potential orthologue of *Arabidopsis* FT florigen that integrates both endogenous and photoperiod flowering signals in maize. J. Exp. Bot. 62, 4833–4842. 21730358 10.1093/jxb/err129 PMC3192997

[pbi13429-bib-0025] Lee, E.A. and Tollenaar, M. (2007) Physiological basis of successful breeding strategies for maize grain yield. Crop Sci. 47, S‐202–S‐215.

[pbi13429-bib-0026] Leivar, P. , Tepperman, J.M. , Cohn, M.M. , Monte, E. , Al‐Sady, B. , Erickson, E. and Quail, P.H. (2012) Dynamic antagonism between phytochromes and PIF family basic helix‐loop‐helix factors induces selective reciprocal responses to light and shade in a rapidly responsive transcriptional network in *Arabidopsis* . Plant Cell, 24, 1398–1419. 22517317 10.1105/tpc.112.095711 PMC3398554

[pbi13429-bib-0027] Li, J. , Li, G. , Wang, H. and Wang Deng, X. (2011) Phytochrome signaling mechanisms. Arabidopsis Book, 9, e0148. 22303272 10.1199/tab.0148 PMC3268501

[pbi13429-bib-0028] Li, L. , Ljung, K. , Breton, G. , Schmitz, R.J. , Pruneda‐Paz, J. , Cowing‐Zitron, C. , Cole, B.J. *et al*. (2012) Linking photoreceptor excitation to changes in plant architecture. Genes. Dev. 26, 785–790. 22508725 10.1101/gad.187849.112 PMC3337452

[pbi13429-bib-0029] Lorrain, S. , Allen, T. , Duek, P.D. , Whitelam, G.C. and Fankhauser, C. (2008) Phytochrome‐mediated inhibition of shade avoidance involves degradation of growth‐promoting bHLH transcription factors. Plant J. 53, 312–323. 18047474 10.1111/j.1365-313X.2007.03341.x

[pbi13429-bib-0030] Mansfield, B.D. and Mumm, R.H. (2014) Survey of plant density tolerance in U.S. maize germplasm. Crop Sci. 54, 157.

[pbi13429-bib-0031] Matsuoka, Y. , Vigouroux, Y. , Goodman, M.M. , Sanchez G. J. , Buckler, E. and Doebley, J. (2002) A single domestication for maize shown by multilocus microsatellite genotyping. Proc. Natl. Acad. Sci. USA, 99, 6080–6084. 11983901 10.1073/pnas.052125199 PMC122905

[pbi13429-bib-0032] Meng, X. , Muszynski, M.G. and Danilevskaya, O.N. (2011) The *FT*‐like *ZCN8* gene functions as a floral activator and is involved in photoperiod sensitivity in maize. Plant Cell, 23, 942–960. 21441432 10.1105/tpc.110.081406 PMC3082274

[pbi13429-bib-0033] Miller, T.A. , Muslin, E.H. and Dorweiler, J.E. (2008) A maize *CONSTANS*‐like gene, *conz1*, exhibits distinct diurnal expression patterns in varied photoperiods. Planta, 227, 1377–1388. 18301915 10.1007/s00425-008-0709-1

[pbi13429-bib-0034] Mizuno, T. , Oka, H. , Yoshimura, F. , Ishida, K. and Yamashino, T. (2015) Insight into the mechanism of end‐of‐day far‐red light (EODFR)‐induced shade avoidance responses in *Arabidopsis thaliana* . Biosci. Biotechnol. Biochem. 79, 1987–1994. 26193333 10.1080/09168451.2015.1065171

[pbi13429-bib-0035] Monte, E. , Alonso, J.M. , Ecker, J.R. , Zhang, Y. , Li, X. , Young, J. , Austin‐Phillips, S. *et al*. (2003) Isolation and characterization of *phyC* mutants in Arabidopsis reveals complex crosstalk between phytochrome signaling pathways. Plant Cell, 15, 1962–1980. 12953104 10.1105/tpc.012971 PMC181324

[pbi13429-bib-0036] Multani, D. , Briggs, S. , Chamberlin, M. , Blakeslee, J. , Murphy, A. and Johal, G. (2003) Loss of an MDR transporter in compact stalks of maize *br2* and sorghum *dw3* mutants. Science, 302, 81–84. 14526073 10.1126/science.1086072

[pbi13429-bib-0037] Nishida, H. , Ishihara, D. , Ishii, M. , Kaneko, T. , Kawahigashi, H. , Akashi, Y. , Saisho, D. *et al*. (2013) Phytochrome C is a key factor controlling long‐day flowering in barley. Plant Physiol. 163, 804–814. 24014575 10.1104/pp.113.222570 PMC3793059

[pbi13429-bib-0038] R Core Team (2018) R: A Language and Environment for Statistical Computing. Vienna, Austria: R Foundation for Statistical Computing. https://www.R‐project.org/.

[pbi13429-bib-0039] Reed, J.W. , Nagpal, P. , Poole, D.S. , Furuya, M. and Chory, J. (1993) Mutations in the gene for the red/far‐red light receptor phytochrome B alter cell elongation and physiological responses throughout *Arabidopsis* development. Plant Cell, 5, 147–157. 8453299 10.1105/tpc.5.2.147 PMC160258

[pbi13429-bib-0040] Rockwell, N.C. , Su, Y.‐S. and Lagarias, J.C. (2006) Phytochrome structure and signaling mechanisms. Annu. Rev. Plant Biol. 57, 837–858. 16669784 10.1146/annurev.arplant.56.032604.144208 PMC2664748

[pbi13429-bib-0041] Rutger, J.N. and Mackill, D.J. (2001) Application of Mendelian genetics in rice breeding. In: Rice genetics IV. Proceedings of the fourth international rice genetics symposium. ( Khush, G.S. , Brar, D.S. and Hardy, B. eds), pp. 27–38. Los Banos, Philippines: International Rice Research Institute.

[pbi13429-bib-0042] Saïdou, A.‐A. , Mariac, C. , Luong, V. , Pham, J.‐L. , Bezançon, G. and Vigouroux, Y. (2009) Association studies identify natural variation at PHYC linked to flowering time and morphological variation in pearl millet. Genetics, 182, 899–910. 19433627 10.1534/genetics.109.102756 PMC2710168

[pbi13429-bib-0043] Salvi, S. , Sponza, G. , Morgante, M. , Tomes, D. , Niu, X. , Fengler, K.A. , Meeley, R. *et al*. (2007) Conserved noncoding genomic sequences associated with a flowering‐time quantitative trait locus in maize. Proc. Natl. Acad. Sci. USA, 104, 11376–11381. 17595297 10.1073/pnas.0704145104 PMC2040906

[pbi13429-bib-0044] Sawers, R.J. , Linley, P.J. , Farmer, P.R. , Hanley, N.P. , Costich, D.E. , Terry, M.J. and Brutnell, T.P. (2002) Elongated mesocotyl1, a phytochrome‐deficient mutant of maize. Plant Physiol. 130, 155–163. 12226496 10.1104/pp.006411 PMC166549

[pbi13429-bib-0045] Sharrock, R.A. and Clack, T. (2004) Heterodimerization of type II phytochromes in Arabidopsis. Proc. Natl. Acad. Sci. USA, 101, 11500–11505. 15273290 10.1073/pnas.0404286101 PMC509229

[pbi13429-bib-0046] Sheehan, M.J. , Farmer, P.R. and Brutnell, T.P. (2004) Structure and expression of maize phytochrome family homeologs. Genetics, 167, 1395–1405. 15280251 10.1534/genetics.103.026096 PMC1470959

[pbi13429-bib-0047] Sheehan, M.J. , Kennedy, L.M. , Costich, D.E. and Brutnell, T.P. (2007) Subfunctionalization of *PhyB1* and *PhyB2* in the control of seedling and mature plant traits in maize. Plant J. 49, 338–353. 17181778 10.1111/j.1365-313X.2006.02962.x

[pbi13429-bib-0048] Spielmeyer, W. , Ellis, M.H. and Chandler, P.M. (2002) Semidwarf (*sd‐1*), "green revolution" rice, contains a defective gibberellin 20‐oxidase gene. Proc. Natl. Acad. Sci. USA, 99, 9043–9048. 12077303 10.1073/pnas.132266399 PMC124420

[pbi13429-bib-0049] Sun, J. , Zheng, T. , Yu, J. , Wu, T. , Wang, X. , Chen, G. , Tian, Y. *et al*. (2017) TSV, a putative plastidic oxidoreductase, protects rice chloroplasts from cold stress during development by interacting with plastidic thioredoxin Z. New Phytol. 215, 240–255. 28248438 10.1111/nph.14482

[pbi13429-bib-0050] Takano, M. , Inagaki, N. , Xie, X. , Yuzurihara, N. , Hihara, F. , Ishizuka, T. , Yano, M. *et al*. (2005) Distinct and cooperative functions of phytochromes A, B, and C in the control of deetiolation and flowering in rice. Plant Cell, 17, 3311–3325. 16278346 10.1105/tpc.105.035899 PMC1315371

[pbi13429-bib-0051] Tollenaar, M. and Lee, E.A. (2002) Yield potential, yield stability and stress tolerance in maize. Field Crops Res. 75, 161–169.

[pbi13429-bib-0052] Wang, H. and Wang, H. (2015) Phytochrome signaling: time to tighten up the loose ends. Mol. Plant, 8, 540–551. 25670340 10.1016/j.molp.2014.11.021

[pbi13429-bib-0053] Winkler, R.G. and Helentjaris, T. (1995) The maize Dwarf3 gene encodes a cytochrome P450‐mediated early step in Gibberellin biosynthesis. Plant Cell, 7, 1307. 7549486 10.1105/tpc.7.8.1307 PMC160953

[pbi13429-bib-0054] Woods, D.P. , Ream, T.S. , Minevich, G. , Hobert, O. and Amasino, R.M. (2014) PHYTOCHROME C is an essential light receptor for photoperiodic flowering in the temperate grass, *Brachypodium distachyon* . Genetics, 198, 397–408. 25023399 10.1534/genetics.114.166785 PMC4174950

[pbi13429-bib-0055] Wu, G. , Zhao, Y. , Shen, R. , Wang, B. , Xie, Y. , Ma, X. , Zheng, Z. *et al*. (2019) Characterization of maize phytochrome‐interacting factors in light signaling and photomorphogenesis. Plant Physiol. 181, 789–803. 31350363 10.1104/pp.19.00239 PMC6776846

[pbi13429-bib-0056] Xiao, C. , Chen, F. , Yu, X. , Lin, C. and Fu, Y.‐F. (2009) Over‐expression of an AT‐hook gene, *AHL22*, delays flowering and inhibits the elongation of the hypocotyl in *Arabidopsis thaliana* . Plant Mol. Biol. 71, 39–50. 19517252 10.1007/s11103-009-9507-9

[pbi13429-bib-0057] Xie, Y. , Liu, Y. , Wang, H. , Ma, X. , Wang, B. , Wu, G. and Wang, H. (2017) Phytochrome‐interacting factors directly suppress MIR156 expression to enhance shade‐avoidance syndrome in *Arabidopsis* . Nat. Commun. 8, 348. 28839125 10.1038/s41467-017-00404-y PMC5570905

[pbi13429-bib-0058] Yang, Q. , Li, Z. , Li, W. , Ku, L. , Wang, C. , Ye, J. , Li, K. *et al*. (2013) CACTA‐like transposable element in *ZmCCT* attenuated photoperiod sensitivity and accelerated the postdomestication spread of maize. Proc. Natl. Acad. Sci. USA, 110, 16969–16974. 24089449 10.1073/pnas.1310949110 PMC3801022

[pbi13429-bib-0059] Yu, H.Q. , Sun, F.A. , Lu, F.Z. , Feng, W.Q. , Zhang, Y.Y. , Liu, B.L. , Yan, L. *et al*. (2018) Positive regulation of phytochrome a on shade avoidance in maize. Pakistan J. Botany, 50, 1433–1440.

[pbi13429-bib-0060] Zhang, Y. , Mayba, O. , Pfeiffer, A. , Shi, H. , Tepperman, J.M. , Speed, T.P. and Quail, P.H. (2013) A quartet of PIF bHLH factors provides a transcriptionally centered signaling hub that regulates seedling morphogenesis through differential expression‐patterning of shared target genes in *Arabidopsis* . PLoS Genet. 9, e1003244. 23382695 10.1371/journal.pgen.1003244 PMC3561105

[pbi13429-bib-0061] Zhao, Y. , Zhang, C. , Liu, W. , Gao, W. , Liu, C. , Song, G. , Li, W.X. *et al*. (2016) An alternative strategy for targeted gene replacement in plants using a dual‐sgRNA/Cas9 design. Sci. Rep. 6, 23890. 27033976 10.1038/srep23890 PMC4817149

[pbi13429-bib-0062] Zheng, T. , Sun, J. , Zhou, S. , Chen, S. , Lu, J. , Cui, S. , Tian, Y. *et al*. (2019) Post‐transcriptional regulation of Ghd7 protein stability by phytochrome and OsGI in photoperiodic control of flowering in rice. New Phytol. 224, 306–320. 31225911 10.1111/nph.16010

