# Supplementary material for: CRISPR/Cas9‐mediated knockout and overexpression studies reveal a role of maize phytochrome C in regulating flowering time and plant height
Source: Plant Biotechnol J. 2020 Jul 2;18(12):2520–32. doi: 10.1111/pbi.13429 (PMC7680541; doi:10.1111/pbi.13429)
Supplement: Supplementary file 1 — Figure S1 Multiple sequence alignment of the protein sequences of ZmPHYC1, ZmPHYC2, OsPHYC and AtPHYC. Figure S2 A heat map illustrating the expression levels of the ZmPHYC genes in different tissues from various developmental stages. Figure S3 ZmPHYC1 and ZmPHYC2 proteins are localized in both the nucleus and cytoplasm in N. benthamiana epidermal cells under light conditions. Figure S4 ZmPHYCs can interact with themselves and ZmPHYBs in plant cells. Figure S5 Expression analysis of the ZmPHYC transgenes in the selected Arabidopsis transgenic lines. Figure S6 ZmPHYC1‐ and ZmPHYC2‐overexpression plants in the phyC‐2 mutant background shows accelerated flowering compared with the phyC‐2 mutant. Figure S7 Fluence‐rate response curves for hypocotyl length under Rc, Bc and FRc light conditions. Figure S8 The zmphyC1 zmphyC2 double knockout mutants do not show an early‐flowering phenotype under natural SD conditions. Figure S9 Transcript profiles of ZmCCT9 and ZmCCA1 genes in the wild type (blue lines) and zmphyC1 zmphyC2 double mutant (red lines). Table S1 Primers used in this study. [file PBI-18-2520-s001.docx]

**Supporting Information**

**Figure S1** Multiple sequence alignment of the protein sequences of ZmPHYC1, ZmPHYC2, OsPHYC and AtPHYC.

**Figure S2** A heat map illustrating the expression levels of the *ZmPHYCs* genes in different tissues from various developmental stages.

**Figure S3** ZmPHYC1 and ZmPHYC2 proteins are localized in both the nucleus and cytoplasm in *N. benthamiana* epidermal cells under light conditions.

**Figure S4** ZmPHYCs can interact with themselves and ZmPHYBs in plant cells.

**Figure S5** Expression analysis of the *ZmPHYCs* transgenes in the selected Arabidopsis transgenic lines.

**Figure S6** *ZmPHYC1-* and *ZmPHYC2*-overexpression plants in the *phyC-2* mutant background shows accelerated flowering compared with the *phyC-2* mutant.

**Figure S7** Fluence-rate response curves for hypocotyl length under Rc, Bc and FRc light conditions.

**Figure S8** The *zmphyC1 zmphyC2* double knockout mutants do not show an early flowering phenotype under natural SD conditions.

**Figure S9** Transcript profiles of *ZmCCT9* and *ZmCCA1* genes in the wild type (blue lines) and *zmphyC1 zmphyC2* double mutant (red lines).

**Table S1** Primers used in this study.


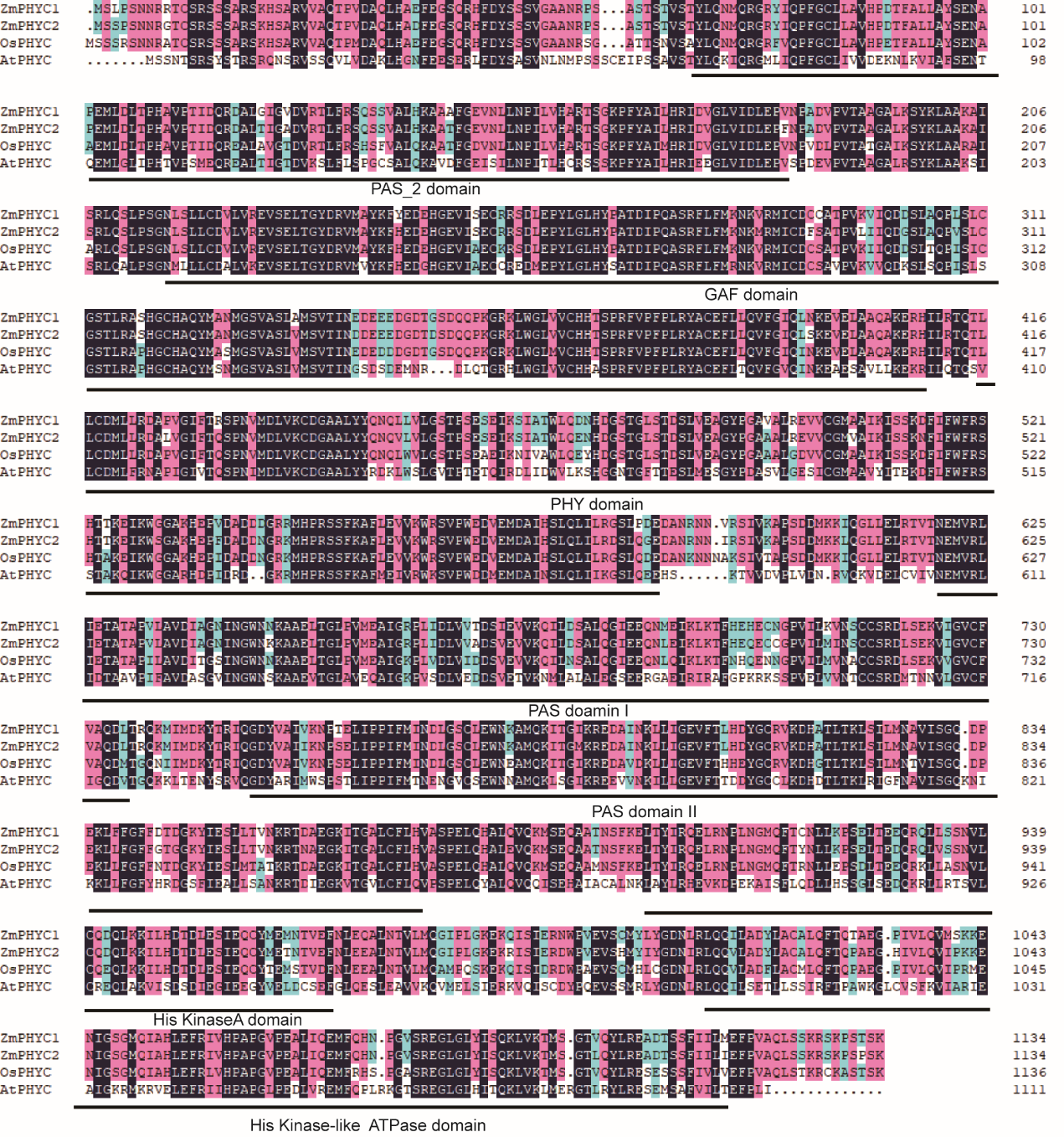


**Figure S1** Multiple sequence alignment of the protein sequences of ZmPHYC1, ZmPHYC2, OsPHYC and AtPHYC. The alignment result is shown by the DNAMAN software (Lynnon Corp, Canada, version 8.0.8). The conserved domains are underlined.


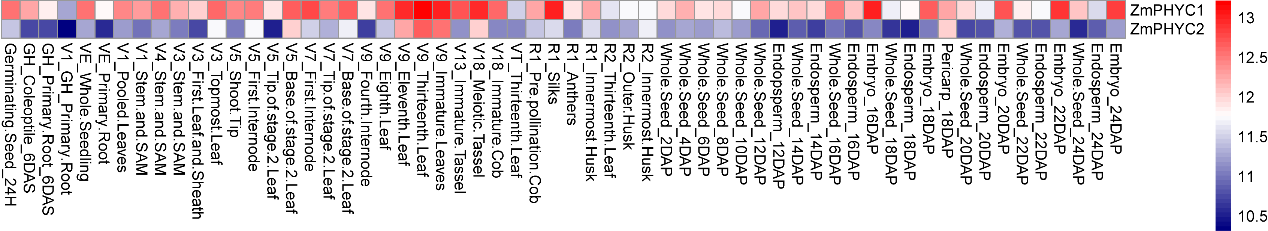


**Figure S2** A heat map illustrating the expression levels of the *ZmPHYCs* genes in different tissues from various developmental stages. The expression heatmap was constructed for the two *ZmPHYCs* genes using transcriptomic data from the maize inbred line B73 ([Sekhon *et al.*, 2011](#_ENREF_39)). Normalized gene expression values are shown in different colors that represent the levels of expression indicated by the scale bar. The red color represents the relatively higher expression level.


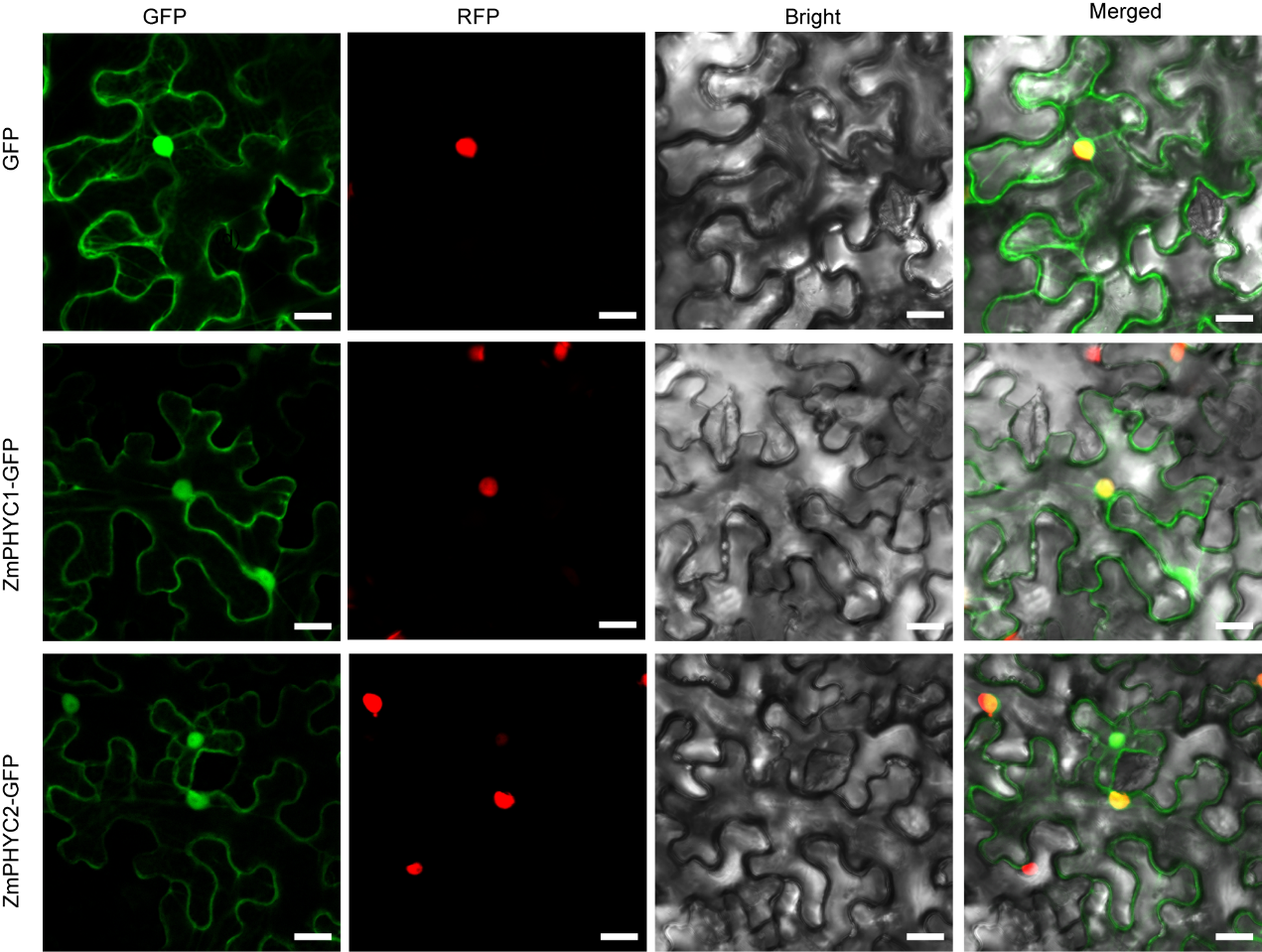


**Figure S3** ZmPHYC1 and ZmPHYC2 proteins are localized in both the nucleus and cytoplasm in *N. benthamiana* epidermal cells under light conditions. The *N. benthamiana* leaves were incubated in darkness for 24 h after transformation and then transferred to a greenhouse (16 h light/8 h dark) for 24 to 36 h before imaging using a confocal microscope. The expression of *Pro-35S::mRFP-AHL22* was used to show the position of the nucleus. The *pCambia1305-35S::GFP* vector serves as a control. Scale bars = 20 µm.


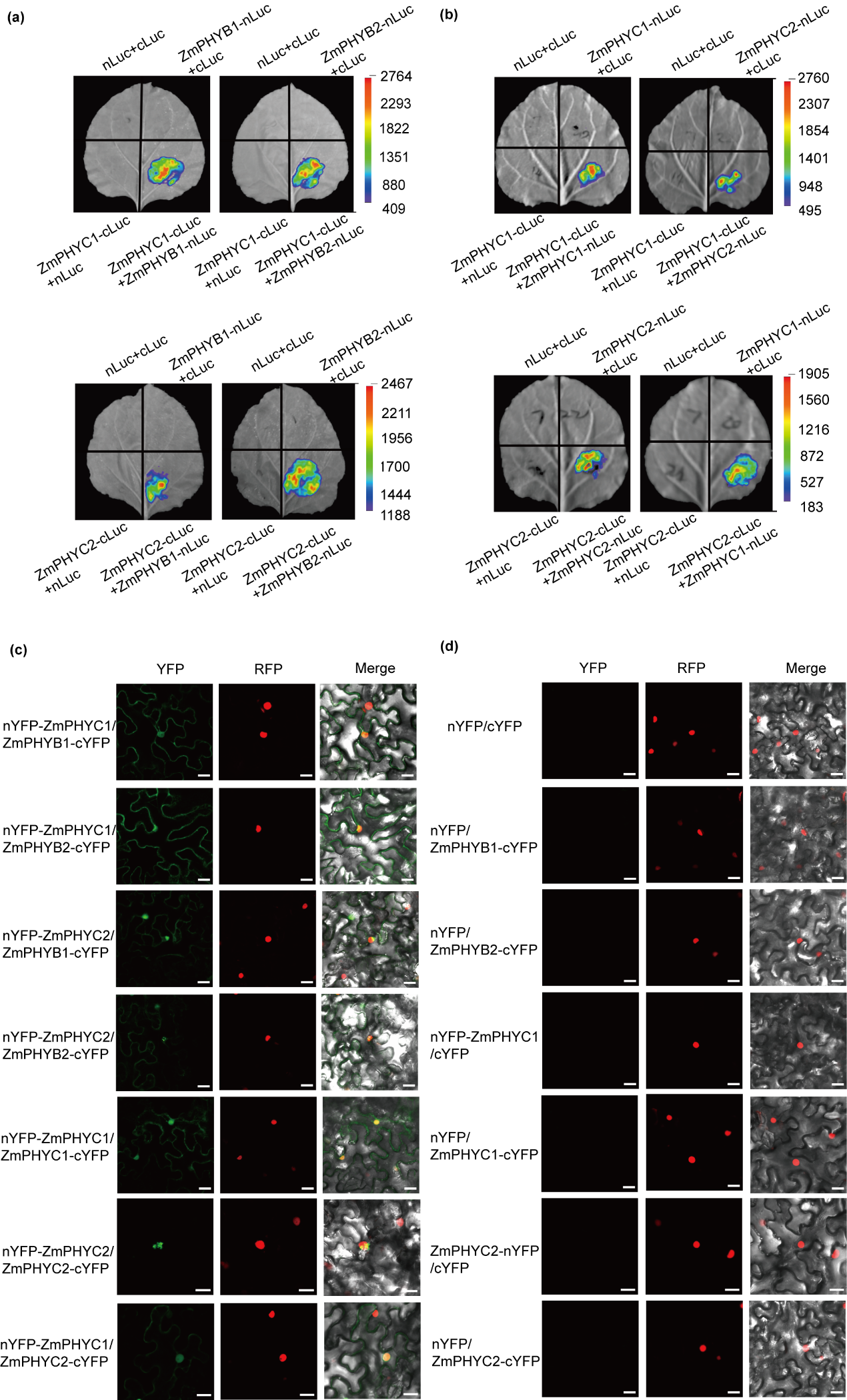


**Figure S4** ZmPHYCs can interact with themselves and ZmPHYBs in plant cells. The *N. benthamiana* leaves were infiltrated with the indicated plasmid combinations, incubated for 24 h in the dark, and then transferred to a greenhouse (16 h light /8 h dark) for 24 to 36 h prior to photographing using an in vivo imaging system (for LCI) or a confocal microscope (for BiFC). (a and b) LCI assay showing the interactions of ZmPHYCs with ZmPHYB members (a) or ZmPHYC members (b) in *N. benthamiana* leaf cells. The full-lengths of *ZmPHYC1* and *ZmPHYC2* were fused to cLuc and nLuc. The full-lengths of ZmPHYBs members were fused to nLuc. (c) BiFC assay showing the interactions of ZmPHYCs with themselves or ZmPHYB members in *N. benthamiana* leaf cells. Full-length ZmPHYC1 and ZmPHYC2 proteins were fused to the split N-terminal fragment of yellow fluorescent protein (nYFP) and the C-terminal fragment of YFP (cYFP). ZmPHYB1 and ZmPHYB2 proteins were fused to the split C-terminal fragment of YFP (cYFP). Bars = 20 μm. (d) Negative controls for BiFC assay. Bars = 20 μm.

**
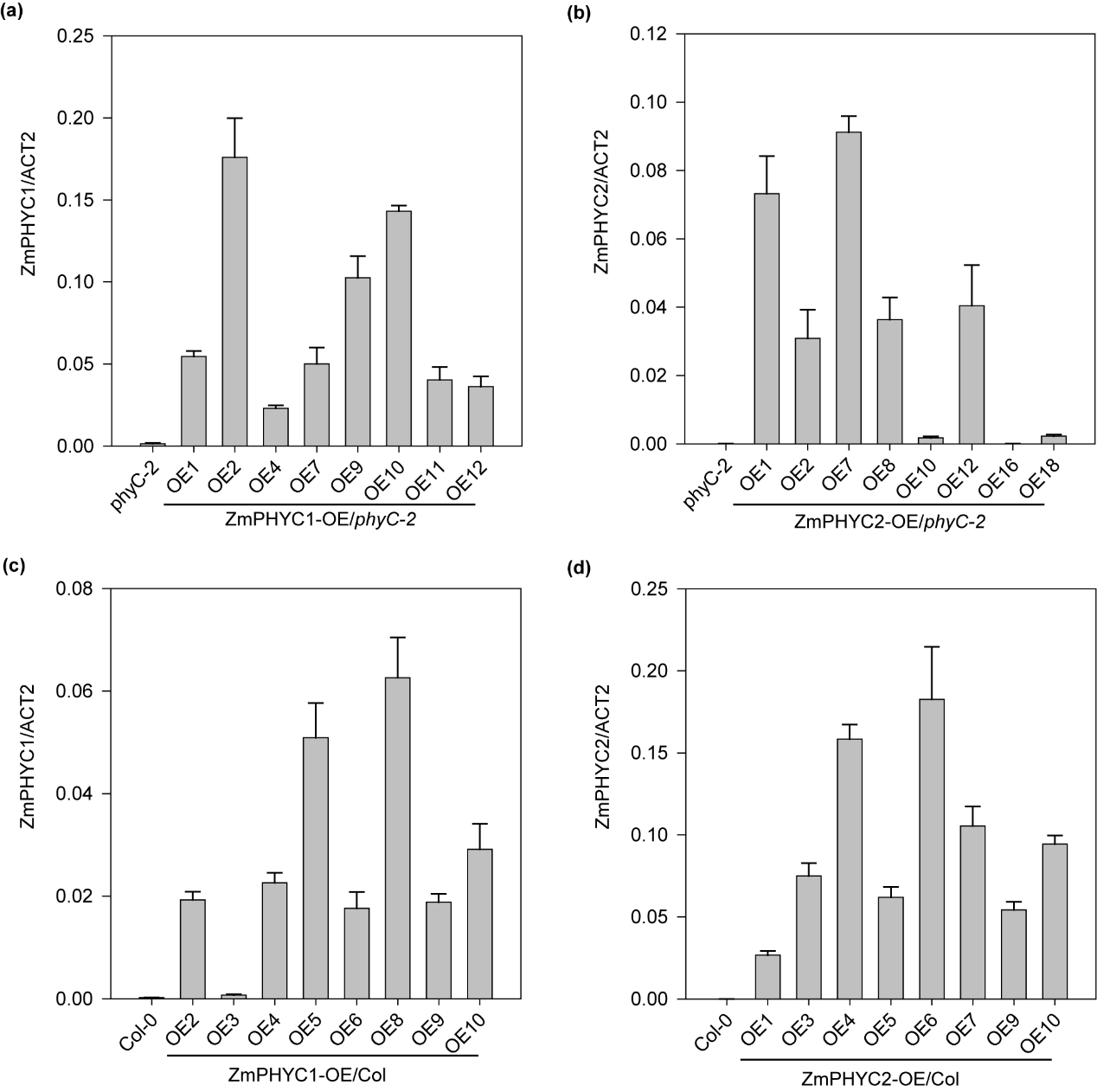
**

**Figure S5** Expression analysis of the *ZmPHYCs* transgenes in the selected Arabidopsis transgenic lines. Expression analysis of *ZmPHYC1* and *ZmPHYC2* transgenic lines in the Arabidopsis *phyC-2* mutant background (a and b) or wild-type Col-0 background (c and d). 7-day-old seedlings were used to perform RT-qPCR analysis. *ACT2* was used as the internal control. Data are means and SD of three independent biological replicates.

**
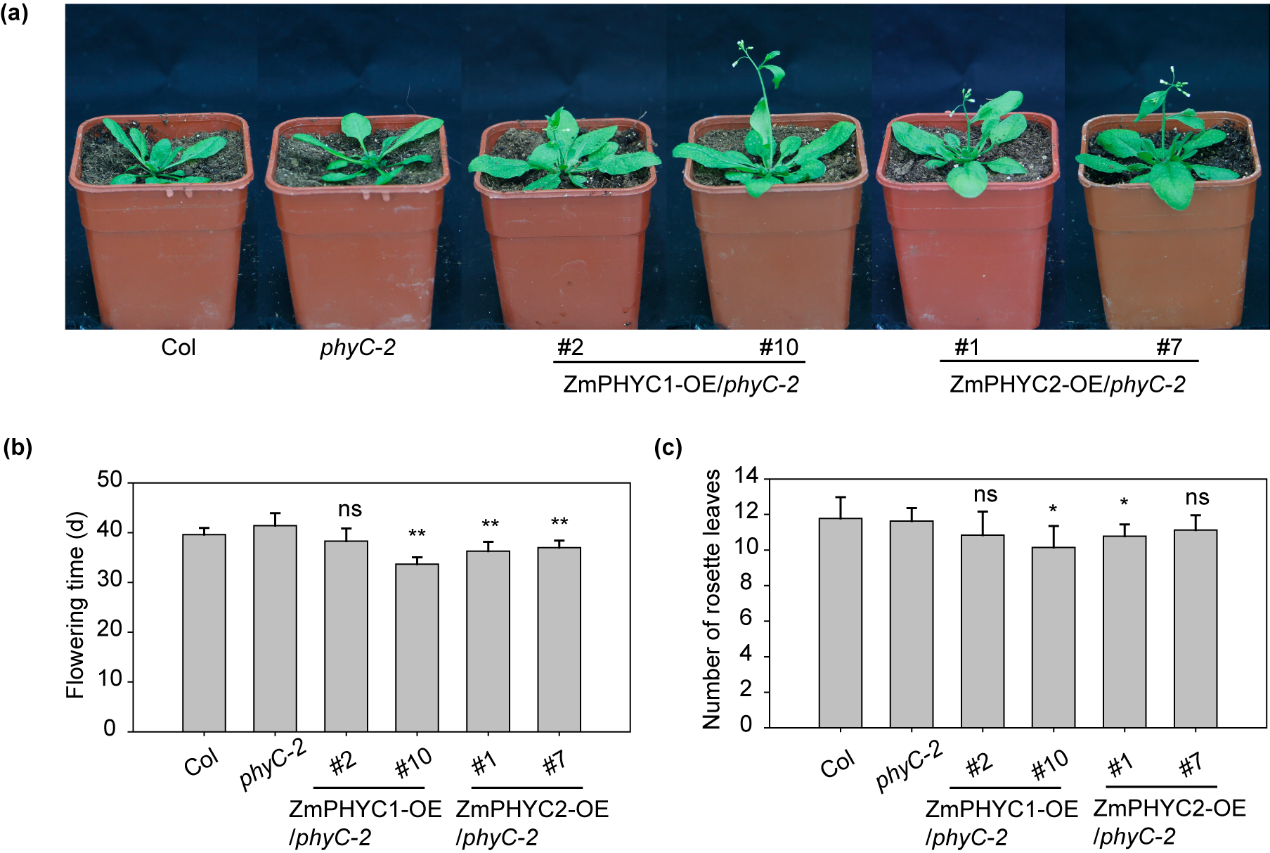
**

**Figure S6** *ZmPHYC1-* and *ZmPHYC2*-overexpression plants in the *phyC-2* mutant background shows accelerated flowering compared with the *phyC-2* mutant. (a) Flowering time phenotype of Col, *phyC-2* and two independent lines of *ZmPHYCs-OE/phyC-2* transgenic plants under LD conditions. (b and c) Flowering time (b) and rosette leaf numbers (c) at flowering for plants in (a). Plants in (a) were grown for 32 d under LD conditions. Data in (b and c) are shown as mean ± SD. Statistical significance analyses were performed between the transgenic plants and *phyC-2* mutant plants according to student’s *t*-test. ∗∗ *p* < 0.01, ∗ *p* < 0.05. ns: Not significant.


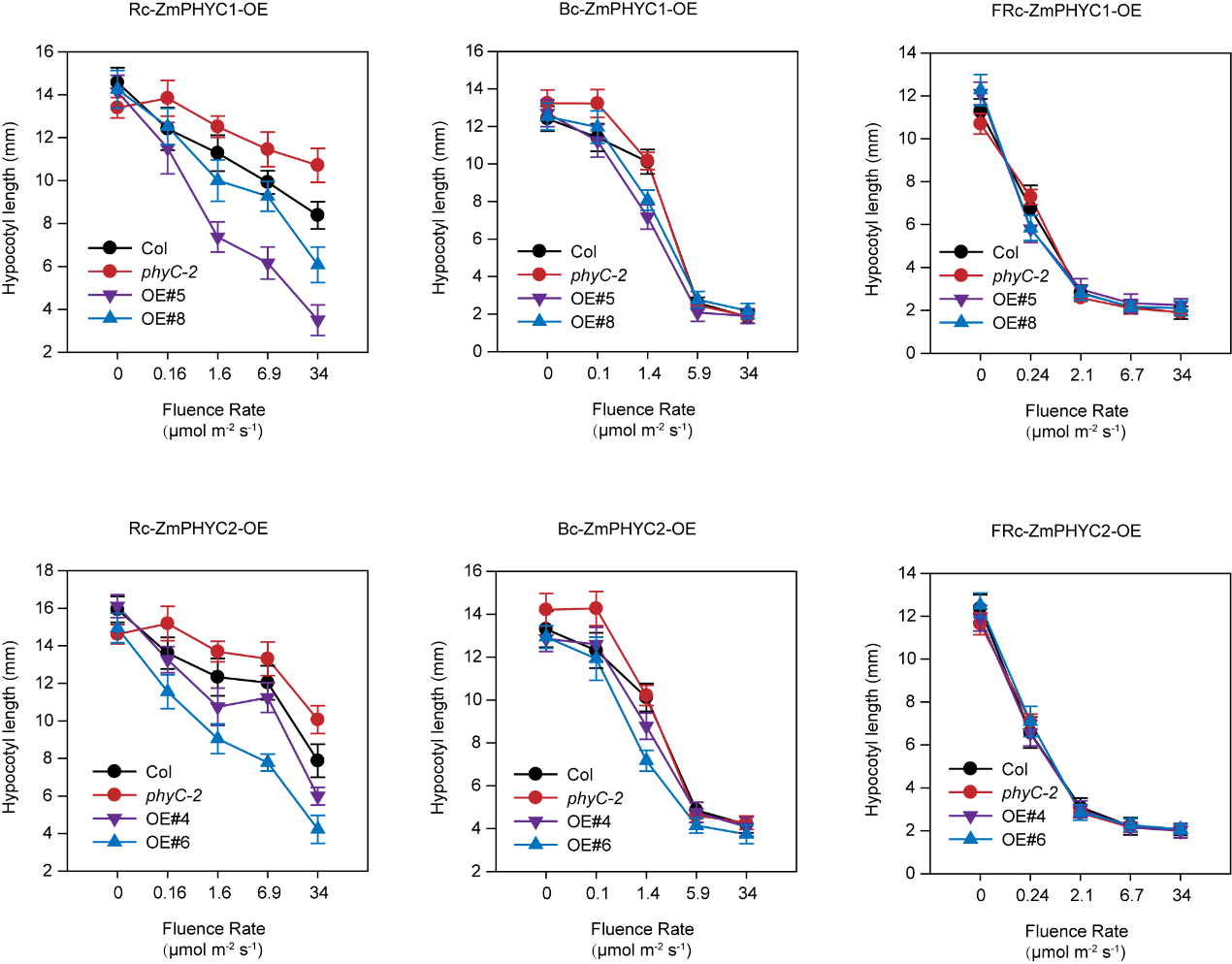


**Figure S7** Fluence-rate response curves for hypocotyl length under continuous Red (Rc), continuous blue (Bc) and continuous far-red (FRc) light conditions. Col-0, *phyC-2* mutant and Col-0 seedlings expressing *ZmPHYC1* and *ZmPHYC2*. Error bar is SD (n = 20).


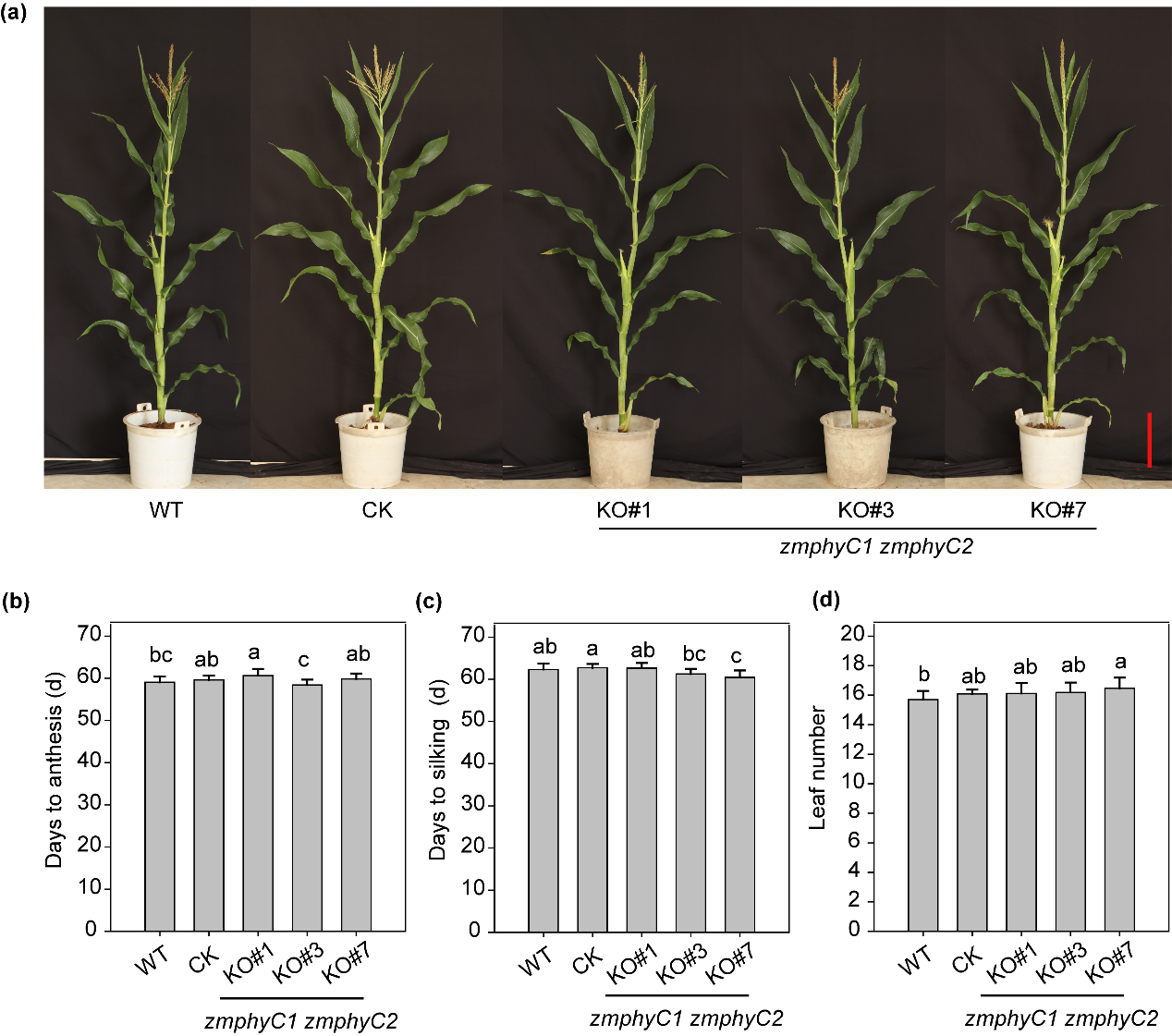


**Figure S8** The *zmphyC1 zmphyC2* double knockout mutants do not show an early flowering phenotype under natural SD conditions. (a) Gross morphologies of WT, CK (null segregates with wild-type *ZmPHYC1* and *ZmPHYC2* genes) and *zmphyC1 zmphyC2* double knockout lines at flowering (anthesis) under natural SD conditions. Bar = 25 cm. (b-d) Days to anthesis (b), days to silking (c) and Leaf number (d) in WT, CK and three knockout lines under natural SD conditions. Data represent the mean and SD from at least 10 maize plants. Different letters indicate significant differences (*p* < 0.05) according to Bonferroni corrected.


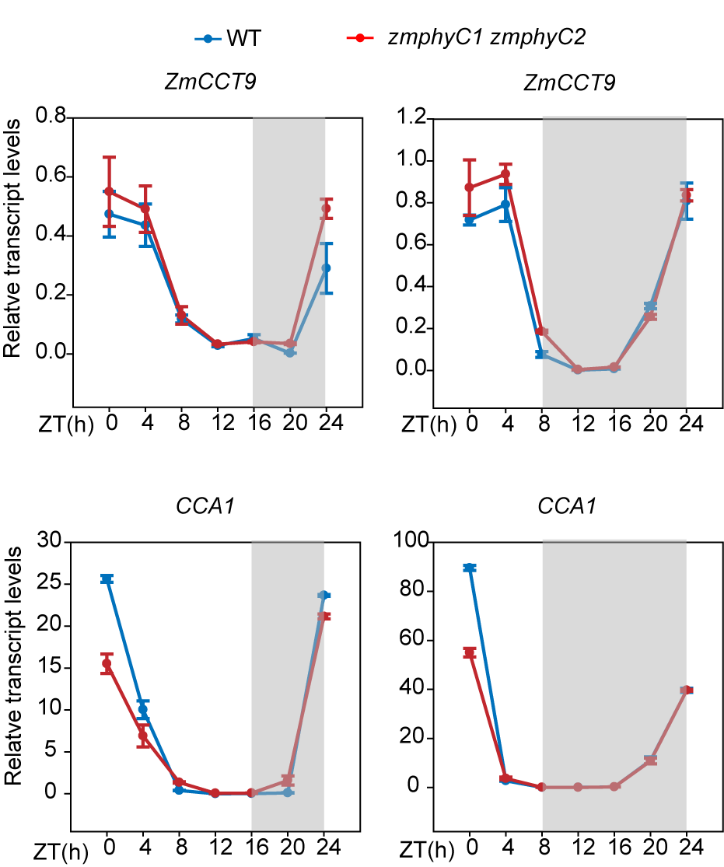


**Figure S9** Transcript profiles of *ZmCCT9* and *ZmCCA1* genes in the wild type (blue lines) and *zmphyC1 zmphyC2* double mutant (red lines). The data are relative to the control gene *Tubulin 5* and represent means ± SD of three biological replicates. Plants were grown under artificial LD and SD conditions. ZT, zeitgeber time. The gray shadows indicate the dark period.

**Supplemental Table 1 Primers used in this study.**

| Primer name | Sequence (5'-3') |
| --- | --- |
| For *ZmPHYC* genes cloning and plant transformation | |
| ZmPHYC1-ubi-F | GTTTGGTGTTACTTCTGCAGATGTCGTTGCCGTCGAAC |
| ZmPHYC1-ubi-R | TGCCACCACCGGATCCGAATTTACTCGTCGAAGGCTT |
| ZmPHYC2-ubi-F | GTTTGGTGTTACTTCTGCAGATGTCGTCGCCGTCGAACAACC |
| ZmPHYC2-ubi-R | TGCCACCACCGGATCCGAATTTACTTGGCGAAGGCTTGGACC |
| For qRT-PCR | |
| ZmPHYC1-qF | TGAGCATGGCGAGGTCATTT |
| ZmPHYC1-qR | GGCTGAGAGGTTGTGCTAGG |
| ZmPHYC2-qF | AGGAGTGTTGTGGTCCAGTT |
| ZmPHYC2-qR | GCCTGGTCAAATCTTGTGCT |
| Tubllin 5-qF | GCCGTTGCCGAGGTGTTC |
| Tubllin 5-qR | GTCCTTCTCAAGAGCAGCCAAGT |
| HFR1-qF | TAAATTGGCCATTACCACCGTTTA |
| HFR1-qR | ACCGTGAAGAG ACTGAGGAGAAGA |
| YUC2-qF | GGAGGAGACGAGACAGGATAAA |
| YUC2-qR | AGCGACTTGAGGTACTCGAT |
| HB2-qF | GAGGTAGACTGCGAGTTCTTACG |
| HB2-qR | GCATGTAGAACTGAGGAGAGAGC |
| PIL1-qF | AAATTGCTCTCAGCCATTCGTGG |
| PIL1-qR | TTCTAAGTTTGAGGCGGACGCAG |
| ACT2-qF | AAATCACAGCACTTGCACCAAGC |
| ACT2-qR | GGCCTTGGAGATCCACATCTGC |
| ZmCCT9-qF | CCACAATCATGTCCTTCTG |
| ZmCCT9-qR | GTAGCGAATCTGCTTCTC |
| ZmCONZ1-qF | GGCTCTGTCGTCTGATGGTAG |
| ZmCONZ1-qR | TCACTCATTACCATCGGTTTCCT |
| ZmCCA1-qF | ATTTGGCTGCTCTTTGGGTC |
| ZmCCA1-qR | ATCTCCAACCAAACGCTGGA |
| ZCN8-qF | TACAACAATAGGCTACTTCT |
| ZCN8-qR | GAATATCTGTCACCATCCA |
| For subcellular localization | |
| ZmPHYC1-GFP-F | CGGAGCTAGCTCTAGAATGTCGTTGCCGTCGAACA |
| ZmPHYC1-GFP-R | TCGAGACGTCTCTAGAGAATTTACTCGTCGAAGGCTTGG |
| ZmPHYC2-GFP-F | CGGAGCTAGCTCTAGAATGTCGTCGCCGTCGAAC |
| ZmPHYC2-GFP-R | TCGAGACGTCTCTAGAGAATTTACTTGGCGAAGGCTTG |
| For LCI assay | |
| ZmPHYB1-nLUC-F | CGGGGGACGAGCTCGGTACCATGGCGTCGGGCAGCCGCG |
| ZmPHYB1-nLUC-R | ACGAGATCTGGTCGACGACGATTTCTCTACCAGCTGCTGGA |
| ZmPHYB2-nLUC-F | CGGGGGACGAGCTCGGTACCATGGCGTCGGACAGTCGC |
| ZmPHYB2-nLUC-R | ACGAGATCTGGTCGACACATATCAGCTGATTTTCTCTACCAGC |
| ZmPHYC1-nLUC-F | CGGGGGACGAGCTCGGTACCATGTCGTTGCCGTCGAACA |
| ZmPHYC1-nLUC-R | ACGAGATCTGGTCGACGAATTTACTCGTCGAAGGCTTGG |
| ZmPHYC2-nLUC-F | CGGGGGACGAGCTCGGTACCATGTCGTCGCCGTCGAAC |
| ZmPHYC2-nLUC-R | ACGAGATCTGGTCGACGAATTTACTTGGCGAAGGCTTG |
| ZmPHYC1-cLUC-F | ACGCGTCCCGGGGCGGTACCATGTCGTTGCCGTCGAACA |
| ZmPHYC1-cLUC-R | AGCTCTGCAGGTCGACTCAGAATTTACTCGTCGAAGGCT |
| ZmPHYC2-cLUC-F | ACGCGTCCCGGGGCGGTACCATGTCGTCGCCGTCGAAC |
| ZmPHYC2-cLUC-R | AGCTCTGCAGGTCGACTCAGAATTTACTTGGCGAAGGC |
| For BiFC assay | |
| p2YC-ZmPHYB1-F | CATTTACGAACGATAGTTAATTAAATGGCGTCGGGCAGCC |
| p2YC-ZmPHYB1-R | CACTGCCACCTCCTCCACTAGTGACGATTTCTCTACCAGCTGCTG |
| p2YC-ZmPHYB2-F | CATTTACGAACGATAGTTAATTAAATGGCGTCGGACAGTCG |
| p2YC-ZmPHYB2-R | CACTGCCACCTCCTCCACTAGTACATATCAGCTGATTTTCTCTACCA |
| p2YC-ZmPHYC1-F | CATTTACGAACGATAGTTAATTAAATGTCGTTGCCGTCGAACA |
| p2YC-ZmPHYC1-R | CACTGCCACCTCCTCCACTAGTGAATTTACTCGTCGAAGGCTTGG |
| p2YC-ZmPHYC2-F | CATTTACGAACGATAGTTAATTAAATGTCGTCGCCGTCGAAC |
| p2YC-ZmPHYC2-R | CACTGCCACCTCCTCCACTAGTGAATTTACTTGGCGAAGGCTTG |
| p2YN-ZmPHYC1-F | CATTTACGAACGATAGTTAATTAAATGTCGTTGCCGTCGAACA |
| p2YN-ZmPHYC1-R | CACTGCCACCTCCTCCACTAGTGAATTTACTCGTCGAAGGCTTGG |
| p2YN-ZmPHYC2-F | CATTTACGAACGATAGTTAATTAAATGTCGTCGCCGTCGAAC |
| p2YN-ZmPHYC2-R | CACTGCCACCTCCTCCACTAGTGAATTTACTTGGCGAAGGCTTG |
| For constructing *zmphyC1 zmphyC2* knock-out mutants * | |
| ZmPHYC1C2-sgR-F1 | U6-1-GTTCTGGAGGTAGGTGGAGA-gRNA |
| ZmPHYC1C2-sgR-F2 | U6-1-GCACATCGCACAGCAACGAC-gRNA |
| sgR-R | GGCCAGTGCCAAGCTTAAAAAAAGCACCGACTCG |
| U6-1-1F | TGCACTGCACAAGCTGCTGTTTTTGTTAGCCCCATCG |
| U6-1-2F | TGCTTTTTTTAAGCTGCTGTTTTTGTTAGCCCCATCG |
| For genotyping of the *zmphyC1 zmphyC2* knock-out mutants | |
| ZmPHYC1-geno-F | GTGTATTCCCTCTTCTCCCCCC |
| ZmPHYC1-geno-R | GGCTTGTATGATGGCAGACGAC |
| ZmPHYC2-geno-F | CTCGCTGAAATTCCCTCTTCTT |
| ZmPHYC2-geno-R | GTTGGTCACTGTCGGTATCCC |

Note: * Forward primers contain maize U6-1 promoter adaptor (GAGCCGCAAGCACCGAATT), target site and part of the guide RNA (gRNA) sequences (GTTTTAGAGCTAGAAATAGCAAGTT).
